# α‐Glucosidase and Acetylcholinesterase (AChE) Inhibitory Activities, Larvicidal Potential and GC–MS Analysis of *Endlicheria paniculata*: A Combined Experimental and Molecular Docking Study

**DOI:** 10.1002/cbdv.71881

**Published:** 2026-10-27

**Authors:** Jenifer Priscila de Araujo, Luiz Everson Da Silva, Wanderlei do Amaral, Michele Debiasi Alberton, Ana Helena Loos Moritz, Fernando Cesar Martins Betim, Deise Prehs Montrucchio, Marilis Dallarmi Miguel, Josiane de Fátima Gaspari Dias, Hélcio Silva Dos Santos, Emmanuel Silva Marinho, Márcia Machado Marinho, Jacilene Silva, Obdulio Gomes Miguel

**Affiliations:** ^1^ Department of Pharmacy Postgraduate Program in Pharmaceutical Sciences Federal University of Paraná Curitiba PR Brazil; ^2^ Postgraduate Program in Sustainable Territorial Development Federal University of Paraná Coastal sector Matinhos PR Brazil; ^3^ Department of Chemical Engineering Federal University of Paraná Curitiba PR Brazil; ^4^ Department of Pharmaceutical Sciences Regional University of Blumenau Blumenau SC Brazil; ^5^ Laboratory For Bioprospecting and Monitoring of Natural Resources ‐ LBMRN Ceará State University Fortaleza CE Brazil

**Keywords:** *Aedes aegypti*, enzyme inhibition, essential oil, Lauraceae, molecular docking, sesquiterpenoids

## Abstract

Essential oils from *Endlicheria paniculata* tissues were characterized by GC–MS, revealing a high concentration of sesquiterpenes. Major constituents were amorpha‐4,9‐dien‐2‐ol (41.12%) in leaves, β‐muurola‐4,10(14)‐dien‐1‐ol (43.54%) in wood, and spathulenol (18.56%) in bark. In preliminary single‐concentration screening assays, the leaf oil demonstrated preliminary in vitro inhibitory potential against α‐glucosidase (98.44%) and acetylcholinesterase (AChE) (97.71%), alongside moderate larvicidal activity against *Aedes aegypti* (${\mathrm{LC}}_{50}=61.56\;\mu g\cdot {\mathrm{mL}}^{-1}$). Molecular docking simulations complemented these preliminary biological findings, showing that major leaf constituents, particularly amorpha‐4,9‐dien‐2‐ol and selina‐3,7(11)‐diene, exhibited favorable predicted binding interactions toward *Ae. aegypti* AChE‐1, driven primarily by hydrophobic interactions. Overall, these findings provide theoretical insights and support further investigation of *E. paniculata* volatiles as potential candidates for vector control research and detailed bio‐guided enzyme inhibition studies.

## Graphical abstract

This study investigates the essential oil composition of *E. paniculata* wood, bark, and leaves by GC–MS. Biological evaluations of the leaf essential oil reveal promising larvicidal potential against *Aedes aegypti* and in vitro inhibitory activities against α‐glucosidase and acetylcholinesterase (AChE), supported by molecular docking simulations detailing key target‐binding interactions.

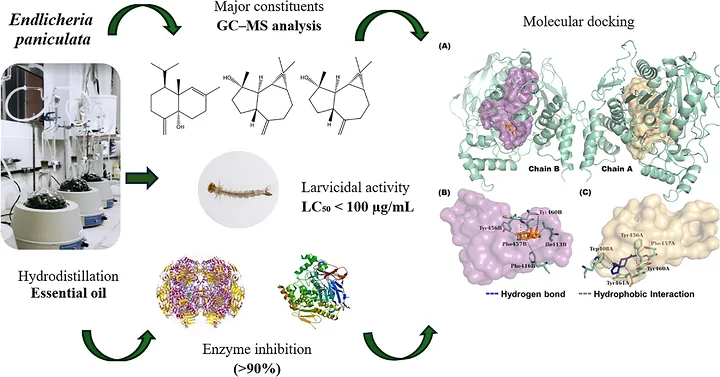

## 1 Introduction

Neurodegenerative diseases are associated with enzyme dysfunctions, which contribute to changes in cellular processes and the accumulation of toxic metabolites [1]. They occur when there is a change (blockage or decrease) in the activities of enzymes that are crucial to the body's metabolic functioning. Acetylcholinesterase (AChE) is a crucial enzyme whose main function is to terminate the neurotransmission caused by the cholinergic motor neurons [2]. Dysfunctions in AChE can cause neurological and neuromuscular problems, such as myasthenia gravis, which causes progressive muscle weakness in different parts of the body, and Parkinson's, which affects the motor system. Furthermore, the prevalence of the disease of cognitive deterioration and memory loss (Alzheimer's) has an increasing trend on the global scenario. It is associated with advanced age, but some conditions modify the risk of Alzheimer's and dementia, such as social context, access to education, excessive alcohol use, hypertension, diabetes, and other factors [3]. In type 2 diabetes, insulin resistance not only impacts peripheral tissues, but also cognitive functions, as insulin resistance impairs the signaling of this anabolic hormone in the brain, contributing to an increased risk of Alzheimer's disease [4]. α‐Glucosidase is a crucial enzyme responsible for catalyzing the final stage of carbohydrate digestion, inhibitors of this enzyme can slow the release of glucose from complex carbohydrates and delay its absorption [5]. The identification of specific inhibitors of enzymes involved in pathological processes allows the development of targeted therapies capable of selectively blocking enzymes. However, synthetic drugs currently employed for the treatment of Alzheimer's disease exhibit significant limitations, including restricted efficacy and the occurrence of adverse effects [6]. Similarly, conventional medications for type 2 diabetes frequently demonstrate important limitations, such as adverse effects, reduced long‐term efficacy, and difficulties in achieving optimal glycemic control [7]. These limitations underscore the need to explore natural alternatives with potentially safer and more sustainable therapeutic profiles. In this context, essential oils have emerged as promising natural agents, offering diverse bioactive properties that can complement or even replace conventional therapies. Essential oils are natural aromatic substances that have been extensively evaluated for their therapeutic effects on the central nervous system. Studies have shown that they can be developed as multipotent agents against neurological disorders, as well as potential neuroprotective remedies for neurodegenerative diseases [8, 9]. These volatile fractions act as versatile components capable of modulating multiple molecular pathways involved in neurodegeneration and aging, suggesting their potential as complementary therapies for Alzheimer's disease and age‐related cognitive decline [8]. In addition to their neuroprotective effects, essential oils have been reported to exhibit bioinsecticidal and larvicidal activities, which further demonstrates their versatile biological potential [10, 11]. In this context, the bioactive compounds may be capable of modifying the neural function and cuticle structure of larvae, leading to their death or interruption of their development. New agents capable of interrupting the life cycle of larvae are relevant to reduce the population of adult mosquitoes and, thus, reduce the transmission chain of vector‐borne diseases. Studies show that essential oils are a promising source for the development of larvicidal materials, being promising alternatives to synthetic insecticides [12]. *A. aegypti* is a mosquito notorious for being a vector of arboviruses, such as Zika, Chikungunya, yellow fever, and the best known of them, dengue. The global distribution of the disease has evolved rapidly over the past three decades, causing health and economic problems in different regions of the world [13]. Effective mosquito control is essential for the prevention of these diseases, as dengue is increasingly establishing itself in large tropical areas, causing 100 to 400 million new infections per year [14].

In this study, we evaluated the biological potential of a species belonging to the Lauraceae family, which has a considerable number of woody plants with relevant economic and ecological value [15]. The *Endlicheria* genus has few effective studies; however, the existing publications are promising. Several species, including *Endlicheria arenosa, Endlicheria bracteolata*, and *E. paniculata*, have demonstrated antimicrobial, antioxidant, antileishmanial, anti‐inflammatory, and antiproliferative activities, highlighting the pharmacological potential and possible therapeutic applications of this genus [16, 17, 18, 19]. Given this background, this study focuses on evaluating the chemical constituents present in the leaves, bark, and wood of the species, as well as the enzymatic inhibition capacity of the essential oil obtained from its dried leaves against α‐glucosidase and AChE. Additionally, the larvicidal activity against third‐instar larvae of *A. aegypti* was evaluated, complemented by molecular docking simulations to elucidate the interactions between the major constituents and the enzymatic targets. This comprehensive approach positions *E. paniculata* as a promising matrix for future research and applications in natural products chemistry.

## 2 Results and Discussion

### 2.1 Analysis of Chemical Composition by GC‐MS and Essential Oil Yield

It was found that *E. paniculata* essential oil presented a yield of 1.8% referring to the crushed dry leaves, while 0.14% was the yield from the bark and 0.08% from the wood. Essential oil yield is an important factor in defining the feasibility of studies of species and their bioactivities [20]. Furthermore, the oil content may vary according to the part of the plant, and the quantity and proportion of chemical constituents. Plants produce significant amounts of complex chemical compounds, which may be relevant in therapeutics [21], which are responsible for the medicinal and aromatic properties of essential oils. Furthermore, the yield and composition of essential oils may be related to different internal and external factors. The biosynthesis of essential oils may vary according to genetic origin, environmental conditions, collection period, and extraction methods [22]. The 1.8% yield for *E. paniculata* leaves is significant for the *Endlicheria* genus, suggesting a high density of secretory structures. The dominance of the amorphane skeleton in leaves, contrasting with muurolane and cadinane types in wood and bark, indicates a highly regulated tissue‐specific biosynthesis. This chemical divergence suggests a metabolic specialization of the species, where different sesquiterpene profiles are synthesized according to the biological role of each plant organ.

Regarding chemical composition, 25 constituents were identified in the essential oils, with a clear predominance of sesquiterpene hydrocarbons and their oxygenated derivatives. Several identified compounds belong to common sesquiterpene skeletons, such as eudesmane, muurolane, and cadinane types, which are frequently reported in the Lauraceae family and may reflect conserved biosynthetic pathways within the genus. The constituents identified in the wood, bark, and leaf samples are presented in Tables 1, 2, 3, respectively. The complete chromatographic parameters, calculated retention indices, and mass spectrometric identification details for all detected constituents across *E. paniculata* tissues are summarized in Table S1.

**TABLE 1:** Chemical constituents of the essential oil from the wood of *E. paniculata*.

N°	Constituents	$R_{t}$ (min)	$RI_{\mathrm{lit}}$	$RI_{\mathrm{calc}}$	Relative area (%) mean ± SD
1	Isobornyl acetate	23.250	1283	1250	4.43 ± 0.27
2	δ‐elemene	25.525	1335	1304	12.34 ± 0.76
3	α‐cubebene	26.020	1348	1316	3.84 ± 0.23
4	β‐elemene	27.860	1389	1361	1.35 ± 0.08
5	γ‐himachalene	31.515	1481	1454	0.81 ± 0.05
6	α‐amorphene	31.615	1483	1456	1.08 ± 0.06
7	δ‐selinene	32.020	1492	1467	1.64 ± 0.10
8	*trans*‐dauca‐4(11),7‐diene	34.405	1556	1530	2.05 ± 0.12
9	Spathulenol	35.540	1577	1561	1.67 ± 0.10
10	β‐copaen‐4‐α‐ol	36.070	1590	1575	3.41 ± 0.21
11	Guaiol	36.340	1600	1583	2.30 ± 0.14
12	Tetradecanal	36.980	1611	1600	6.45 ± 0.41
13	*cis*‐isolongifolanone	37.050	1612	1602	1.63 ± 0.10
14	Isolongifolan‐7‐α‐ol	37.110	1618	1604	0.65 ± 0.06
15	10‐epi‐*γ‐*eudesmol	37.320	1622	1610	3.49 ± 0.21
16	β‐muurola‐4,10(14)‐dien‐1‐ol	37.570	1630	1617	43.54 ± 1.90
17	*cis‐*cadin‐4‐en‐7‐ol	37.700	1635	1621	1.02 ± 0.06
18	*epi‐*α‐muurolol	38.560	1640	1645	2.36 ± 0.14
19	Amorpha‐4,9‐dien‐2‐ol	40.865	1700	1712	0.93 ± 0.05
20	10‐nor‐calamenen‐10‐one	40.930	1702	1713	0.67 ± 0.08
21	γ‐(Z)‐curcumen‐12‐ol	42.125	1728	1749	0.86 ± 0.12
22	*n*‐pentadecanol	43.580	1773	1793	1.11 ± 0.00
23	Cyclopentadecanolide	44.335	1832	1816	1.93 ± 0.12
24	Eudesm‐7(11)‐en‐4‐ol acetate	44.375	1839	1817	0.21 ± 0.01
25	*n‐*pentacosane	66.980	2500	2495	0.23 ± 0.01
**Chemical Class**					**Relative Area (%)**
Monoterpene hydrocarbons					0.00
Oxygenated monoterpenes					4.43
Sesquiterpene hydrocarbons					23.11
Oxygenated sesquiterpenes					62.74
Nonterpenoids/Others					9.72
					**Total identified % 100**

**N°**: Compound number in order of elution on the HP‐5MS column; $R_{t}$: Retention time (minutes); $RI_{\mathrm{calc}}$: Linear retention index calculated on an HP‐5MS capillary column relative to n‐alkanes ($C_{7}-C_{30}$) using the Van den Dool and Kratz equation under the same chromatographic conditions; $RI_{\mathrm{lit}}$: Linear retention index reported in the literature (Adams, 2017); **SD**: Standard deviation from triplicate analyses. Compound identification criteria (MSI Level 2) are detailed in the Experimental section.

**TABLE 2:** Chemical Constituents of the essential oil from the bark of *E. paniculata*.

N°	Constituents	$R_{t}$ (min)	$RI_{\mathrm{lit}}$	$RI_{\mathrm{calc}}$	Relative area (%) mean ± SD
1	Isobornyl acetate	23.215	1283	1250	1.89 ± 0.11
2	δ‐elemene	25.500	1335	1303	10.87 ± 0.75
3	α‐cubebene	26.025	1348	1316	3.55 ± 0.21
4	β‐patchoulene	26.815	1379	1336	2.56 ± 0.15
5	β‐cubebene	27.275	1387	1347	1.13 ± 0.07
6	β‐elemene	27.845	1389	1361	3.00 ± 0.18
7	Aromadendrene	29.955	1439	1413	2.55 ± 0.15
8	α‐himachalene	30.565	1449	1429	3.70 ± 0.22
9	γ‐himachalene	31.520	1481	1454	1.74 ± 0.10
10	γ‐amorphene	32.160	1495	1471	1.78 ± 0.11
11	Bicyclogermacrene	32.325	1500	1485	1.96 ± 0.12
12	*cis‐*muurol‐5‐en‐4‐α‐ol	34.980	1559	1546	0.87 ± 0.05
13	Maaliol	35.275	1566	1554	1.22 ± 0.07
14	Spathulenol	35.600	1577	1563	18.56 ± 1.10
15	Caryophyllene oxide	35.755	1582	1567	1.36 ± 0.08
16	Viridiflorol	35.925	1592	1571	6.66 ± 0.40
17	Carotol	36.205	1594	1579	3.32 ± 0.20
18	Guaiol	36.330	1600	1582	1.90 ± 0.11
19	5*‐epi*‐7‐*epi*‐α‐eudesmol	36.700	1607	1592	1.21 ± 0.08
20	Tetradecanal	36.985	1611	1600	5.69 ± 0.36
21	Isolongifolan‐7‐*α*‐ol	37.120	1618	1604	1.18 ± 0.07
22	10‐*epi‐*γ‐eudesmol	37.305	1622	1609	0.78 ± 0.05
23	1‐*epi*‐cubenol	37.395	1627	1612	5.36 ± 0.34
24	β‐muurola‐4,10(14)‐dien‐1‐ol	37.555	1630	1616	14.52 ± 0.87
25	Allo‐aromadendrene epoxide	37.715	1639	1621	2.64 ± 0.16
**Chemical Class**					**Relative Area (%)**
Monoterpene hydrocarbons					0.00
Oxygenated monoterpenes					1.89
Sesquiterpene hydrocarbons					32.84
Oxygenated sesquiterpenes					59.58
Nonterpenoids/Others					5.69
					**Total identified % 100**

**N°**: Compound number in order of elution on the HP‐5MS column; $R_{t}$: Retention time (minutes); $RI_{\mathrm{calc}}$: Linear retention index calculated on an HP‐5MS capillary column relative to n‐alkanes ($C_{7}-C_{30}$) using the Van den Dool and Kratz equation under the same chromatographic conditions; $RI_{\mathrm{lit}}$: Linear retention index reported in the literature (Adams, 2017); **SD**: Standard deviation from triplicate analyses. Compound identification criteria (MSI Level 2) are detailed in the Experimental section.

**TABLE 3:** Chemical Constituents of the essential oil from the leaves of the *E. paniculata*.

N°	Constituents	$R_{t}$ (min)	$RI_{\mathrm{lit}}$	$RI_{\mathrm{calc}}$	Relative area (%) mean ± SD
1	Sabinene	7.865	969	987	0.75 ± 0.04
2	γ‐elemene	25.480	1434	1403	5.54 ± 0.33
3	Dehydro‐aromadendrane	27.845	1460	1461	0.92 ± 0.05
4	γ‐gurjunene	29.115	1475	1492	1.62 ± 0.10
5	Viridiflorene	29.905	1496	1512	0.77 ± 0.05
6	α‐muurolene	30.055	1500	1516	1.47 ± 0.09
7	γ‐cadinene	30.255	1513	1521	1.12 ± 0.10
8	δ‐cadinene	30.335	1522	1523	0.43 ± 0.03
9	Zonarene	30.630	1528	1531	2.18 ± 0.16
10	*cis*‐calamenene	31.060	1528	1542	0.78 ± 0.06
11	*trans‐*cadina‐1,4‐diene	31.515	1533	1554	3.13 ± 0.21
12	α‐cadinene	31.595	1537	1556	0.83 ± 0.05
13	Selina‐3,7(11)‐diene	32.005	1545	1566	16.21 ± 0.97
14	*trans*‐dauca‐4(11),7‐diene	32.150	1556	1570	3.06 ± 0.20
15	Germacrene B	32.330	1559	1575	2.97 ± 0.18
16	Maaliol	32.660	1566	1583	1.97 ± 0.12
17	Germacrene D‐4‐ol	32.860	1574	1589	1.50 ± 0.09
18	Carotol	33.005	1594	1592	0.66 ± 0.04
19	Guaiol	33.220	1600	1598	0.89 ± 0.05
20	5‐*epi*‐7‐*epi‐*α‐eudesmol	34.165	1607	1624	0.53 ± 0.04
21	10‐*epi‐*γ‐eudesmol	34.980	1622	1646	0.77 ± 0.06
22	β‐muurola‐4,10(14)‐dien‐1‐ol	35.565	1630	1662	9.05 ± 0.58
23	Helifolenol A	36.015	1674	1674	0.82 ± 0.06
24	Eudesm‐7(11)‐en‐4‐ol	37.310	1700	1709	0.91 ± 0.06
25	Amorpha‐4,9‐dien‐2‐ol	37.565	1700	1717	41.12 ± 2.45
**Chemical Class**					**Relative Area (%)**
Monoterpene hydrocarbons					0.75
Oxygenated monoterpenes					0.00
Sesquiterpene hydrocarbons					41.03
Oxygenated sesquiterpenes					58.22
Nonterpenoids/Others					0.00
					**Total identified % 100**

**N°**: Compound number in order of elution on the HP‐5MS column; $R_{t}$: Retention time (minutes); $RI_{\mathrm{calc}}$: Linear retention index calculated on an HP‐5MS capillary column relative to n‐alkanes ($C_{7}-C_{30}$) using the Van den Dool and Kratz equation under the same chromatographic conditions; $RI_{\mathrm{lit}}$: Linear retention index reported in the literature (Adams, 2017); **SD**: Standard deviation from triplicate analyses. Compound identification criteria (MSI Level 2) are detailed in the Experimental section.

In the wood essential oil, β‐muurola‐4,10(14)‐dien‐1‐ol was the major constituent identified in the sample with a mean relative GC‐FID peak area of 43.54%. The other compounds found in higher proportions were: δ‐elemene (12.34%), tetradecanal (6.45%), and isobornyl acetate (4.43%). Furthermore, lower percentages of *β‐*copaen‐4‐α‐ol, epi*‐α‐*muurolol, and Guaiol were also identified. Regarding biological activities, *δ‐*elemene has been reported to exhibit antitumor activity against cervical carcinoma cells [23] and to mitigate oxidative stress and neuronal apoptosis, highlighting potential neuroprotective effects [24]. To date, no biological activities have been reported for *β*‐muurola‐4,10(14)‐dien‐1‐ol, which was also identified as a major constituent in the other tissues. This highlights the need for further studies on this specific metabolite to elucidate its pharmacological role. Tetradecanal, a long‐chain aliphatic aldehyde, has demonstrated antiproliferative and anticancer activities in vitro [25], suggesting its potential role as a bioactive compound.

In the sampled bark, spathulenol was the major constituent identified, with a relative peak area of 18.56%. Other major constituents included β‐muurola‐4,10(14)‐dien‐1‐ol (14.52%), δ‐elemene (10.87%), and viridiflorol (6.66%). Furthermore, lower percentages of *α‐*himachalene, *α‐*cubebene, and carotol were also identified. The compound spathulenol is known to have several therapeutic properties. For instance, one study showed that it can inhibit the ABCB1 efflux pump in cells, suggesting its potential use in combination chemotherapy against multidrug‐resistant lymphomas [26]. Additionally, the neuroprotective efficacy of spathulenol has been verified, raising the possibility of using this compound as a targeting agent in the treatment of neurodegenerative diseases [27]. *Viridiflorol* has been reported to exhibit anticancer activity, and other potential bioactivities include anti‐inflammatory, antioxidant, and antimycobacterial effects [28, 29].

Amorpha‐4,9‐dien‐2‐ol was the major constituent identified in the essential oil from *E. paniculata* leaves, with a relative peak area of 41.12%. Other prominent compounds included seline‐3,7(11)‐diene (16.21%), β‐muurola‐4,10(14)‐dien‐1‐ol (9.05%), and γ‐elemene (5.54%). In addition, lower percentages of germacrene B, zonarene, and maaliol were also identified. To the best of our knowledge, this is the first report highlighting amorpha‐4,9‐dien‐2‐ol as a major component of an active essential oil, as its biological properties remain largely unexplored in the literature. However, the constituent seline‐3,7(11)‐diene, is the second major constituent of the dried leaves of *E. paniculata*, has previously been associated with antimicrobial activity [30].

Chemical composition analysis revealed a predominant presence of sesquiterpenoids across all essential oil samples of *E. paniculata*. Specifically, oxygenated sesquiterpenes constituted the major chemical class in all tissues, accounting for 62.74% in wood, 59.58% in bark, and 58.22% in leaves. Sesquiterpene hydrocarbons represented the second most abundant group, comprising 23.11% in wood, 32.84% in bark, and 41.03% in leaves. In contrast, monoterpenes were present in minor proportions, with leaf oil containing only 0.75% monoterpene hydrocarbons and no oxygenated monoterpenes (0%). These variations in the relative abundance of sesquiterpene classes suggest a tissue‐dependent distribution of secondary metabolites in *E. paniculata*, pointing toward metabolic specialization among leaves, bark, and wood. Because the occurrence of these oxygenated sesquiterpenes as major components has been rarely reported, these findings underscore the novelty of this study and its potential chemosystematic significance. The identification of amorpha‐4,9‐dien‐2‐ol as the primary leaf constituent is a key chemotaxonomic marker. Although the amorphane and cadinane skeletons share the same biosynthetic precursor, the specific cyclization to amorphane is rare in *Endlicheria*. This unique “chemical fingerprint” distinguishes *E. paniculata* from other species like *E. arenosa*, reinforcing the intra‐generic variability common in the Lauraceae family. A study carried out with the species *E. arenosa*, reports a higher quantity of sesquiterpene hydrocarbons (77.6%) in the essential oil sample of the species [16]. Furthermore, in the species *E. bracteolata*, 85.8% of sesquiterpene hydrocarbons were also identified [17].

Sesquiterpene compounds belonging to the terpene class contain 15 carbon atoms (C_15_) and are known for their structural diversity and wide occurrence in plants. Studies have already stated its therapeutic effects, such as anti‐inflammatory, antimicrobial, antineoplastic, gastroprotective, antimycobacterial, and immunomodulatory Properties [31, 32]. Sesquiterpenes, particularly tricyclic and oxygenated types, are widely distributed and have been associated with multiple biological activities. Tricyclic sesquiterpenes exhibit notable anti‐inflammatory, antioxidant, and antibacterial properties [33], while sesquiterpenes predominating in the essential oil of *Teucrium viscidum* (Lamiaceae) have demonstrated potent antimicrobial and larvicidal activities [34]. Furthermore, sesquiterpenes in general constitute one of the most diverse classes of terpenoids, with reported antiproliferative, cytotoxic, and antimicrobial effects [35]. These findings support the link between the dominant sesquiterpene compounds identified in our samples and the bioactivities observed.

This study provides the first detailed report on the chemical composition of essential oils obtained from different parts of *E. paniculata* (wood, bark, and leaves). Due to the low extraction yields obtained from bark and wood tissues, biological evaluations were focused exclusively on the leaf essential oil, which represents a sustainable resource enriched in the unique oxygenated sesquiterpene amorpha‐4,9‐dien‐2‐ol. Figure 1 expresses the chemical structures of the majority constituents of the samples.

**FIGURE 1:**
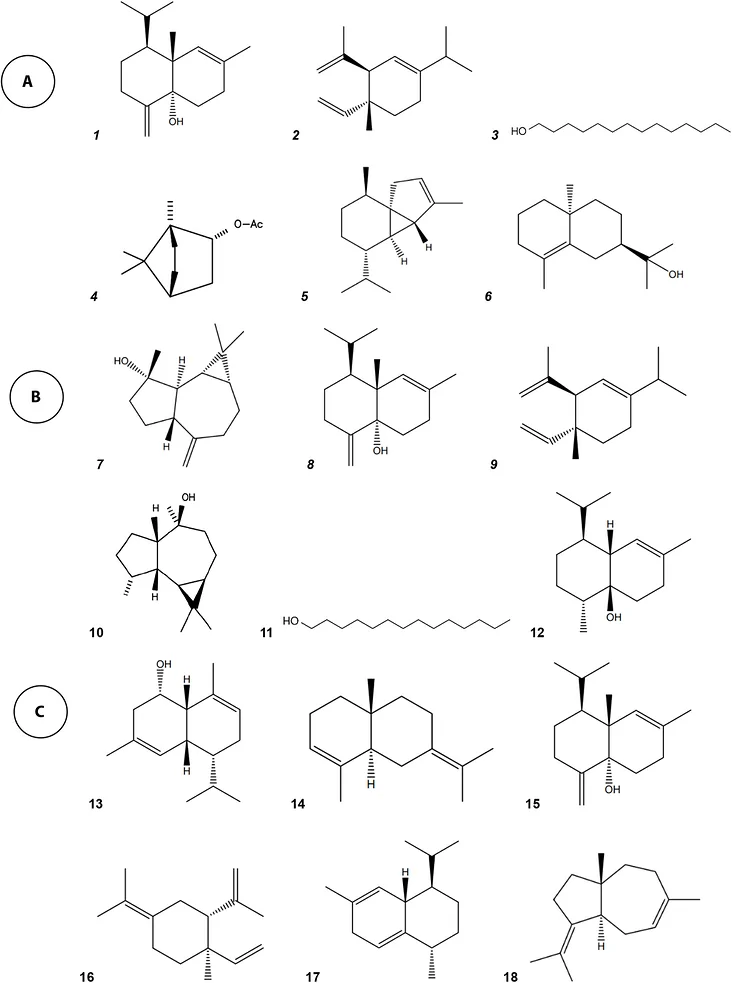
Chemical structures of the main constituents identified in the essential oils of *E. paniculata*. (A) Wood oil constituents 1–6: 1, isobornyl acetate; 2, δ‐elemene; 3, α‐cubebene; 4, β‐elemene; 5, tetradecanal; 6, β‐muurola‐4,10(14)‐dien‐1‐ol. (B) Bark oil constituents 7–12: 7, spathulenol; 8, viridiflorol; 9, carotol; 10, 1‐*epi*‐cubenol; 11, β‐muurola‐4,10(14)‐dien‐1‐ol; 12, tetradecanal. (C) Leaf oil constituents 13–18: 13, γ‐elemene; 14, *trans*‐cadina‐1,4‐diene; 15, selina‐3,7(11)‐diene; 16, *trans*‐dauca‐4(11),7‐diene; 17, β‐muurola‐4,10(14)‐dien‐1‐ol; 18, amorpha‐4,9‐dien‐2‐ol.

### 2.2 *α*‐Glucosidase and Acetylcholinesterase (AChE) Inhibition Activity

In this preliminary single‐concentration screening assay, the essential oil from *E. paniculata* leaves exhibited high inhibitory percentages against both AChE (97.71±0.42%) and α‐glucosidase (98.44±2.71%), as detailed in Table 4.

**TABLE 4:** Preliminary enzyme inhibition screening of *E. paniculata* dry leaf essential oil against acetylcholinesterase (AChE) and α‐glucosidase.

Sample/Group	Assay concentration	Target enzyme	Inhibition activity (%)
Leaf Essential Oil	$50.38\;\mu g\;{\mathrm{mL}}^{-1}$	AChE	$97.71\pm 0.42^{a}$
Neostigmine hydrochloride (Positive Control)	$5.04\;\mu g\;{\mathrm{mL}}^{-1}$	AChE	$100.00\pm 0.00^{b}$
Leaf Essential Oil	$7.19\;\mu g\;{\mathrm{mL}}^{-1}$	α‐Glucosidase	$98.44\pm 2.71^{a}$
Acarbose (Positive Control)	$3.60\;\mu g\;{\mathrm{mL}}^{-1}$	α‐Glucosidase	$100.00\pm 0.00^{b}$

Assay conditions: Results are expressed as mean ± standard deviation (SD) from n=3 independent biological experiments, each performed in technical triplicate. Concentrations represent final nominal test concentrations within the reaction mixture well/tube. Different superscript letters (^a,b^) within the same enzyme category denote statistically significant differences (p<0.001, One‐way ANOVA followed by Tukey's post‐hoc test). The solvent vehicle served as negative control (0% inhibition).

abbreviation: AChE = Acetylcholinesterase.

Regarding inhibitory capacity, samples capable of inhibiting more than 50% of enzymatic activity at initial test concentrations are conventionally considered promising candidates for bio‐guided investigation [36]. Based on this criterion, *E. paniculata* leaf essential oil demonstrates notable preliminary inhibitory potential. While a high inhibition percentage at a single test concentration serves as a valuable primary indicator of biological reactivity, it does not establish pharmacological potency or allow direct quantitative comparison with standard controls (neostigmine and acarbose), which were evaluated at different final concentrations. Detailed concentration–response evaluations to determine $IC_{50}$ values, Hill slopes, and comparative inhibition kinetics against reference standards will be required in future studies following bio‐guided isolation of individual active constituents.

Inhibition of AChE prevents the enzymatic degradation of acetylcholine at synaptic junctions, thereby prolonging neurotransmitter availability. In vitro inhibition of α‐glucosidase, in turn, reflects an ability to delay carbohydrate breakdown. Essential oils represent complex mixtures of volatile terpenes whose lipophilic nature facilitates interactions with active enzyme sites. According to Ayaz et al. (2017), plant volatiles can modulate key enzymes through competitive, noncompetitive, or allosteric hydrophobic interactions [8]. While preliminary in vitro assays provide valuable chemical‐biological insights, extrapolated claims regarding neuroprotection, blood–brain barrier penetration, or clinical management of metabolic disorders remain hypothetical until validated by in vivo pharmacokinetics and full kinetic profiles.

To date, no prior enzyme inhibition studies have been reported for essential oils derived from the genus *Endlicheria*, making these results a novel contribution to the chemistry of the Lauraceae family. However, several related Lauraceae species have shown marked cholinesterase and glycosidase inhibitory properties. For instance, leaf essential oils from *Rhodostemonodaphne parvifolia* [37] and eleven other Amazonian Lauraceae species (including *Endlicheria citriodora*, *Ocotea minor*, *Rhodostemonodaphne negrensis*, and *Aniba panurensis*) demonstrated positive AChE inhibition in preliminary screens [38]. Similarly, *Beilschmiedia madang* leaf oil exhibited 55% AChE inhibition [39].

Regarding α‐glucosidase, *Laurus nobilis* leaf essential oil inhibited the enzyme by over 90% at test screening concentrations, with its isolated compounds (1,8‐cineole, α‐pinene, and limonene) displaying variable individual activities [40]. In quantitative studies, stem and leaf oils of *Neolitsea coccinea* yielded $IC_{50}$ values of $32.2\pm 0.8\;\mu g\;{\mathrm{mL}}^{-1}$ and $70.9\pm 1.1\;\mu g\;{\mathrm{mL}}^{-1}$, respectively [41], while *Apollonias barbujana*, *Laurus novocanariensis*, *Ocotea foetens*, and *Persea indica* demonstrated strong anti‐glucosidase potential [42]. These literary comparisons underscore the pharmacological relevance of volatile secondary metabolites within Lauraceae.

The high primary inhibition (>97%) observed for *E. paniculata* leaf oil is tentatively attributed to its rich lipophilic sesquiterpene fraction, particularly oxygenated derivatives such as *amorpha‐4,9‐dien‐2‐ol*. These C15 architectures feature compact hydrophobic skeletons and polar functional groups capable of anchoring within active site pockets through hydrophobic contact and hydrogen bonding. Further bio‐guided fractionation will be required to confirm whether this activity stems from individual major constituents or synergistic interactions among the volatile mixture.

### 2.3 Larvicidal Activity

The toxicity evaluation demonstrated that *E. paniculata* leaf essential oil exhibits promising larvicidal potential against *A. aegypti* larvae. Initial screening revealed a concentration‐dependent mortality rate after 24 h of exposure, reaching a maximum mortality of 77.50% at the highest tested concentration of 1000 μg/mL, as presented in Table 5.

**TABLE 5:** Larvicidal screening of *E. paniculata* leaf essential oil against *A. aegypti* larvae after 24 h of exposure.

Sample	Concentration (µg/mL)	Mortality (%) ± SD
EpEO Negative Control	1000	77.50 ± 9.57
	100	60.00 ± 8.16
	10	27.50 ± 12.58
		0.00 ± 0.00

EpEO: *E. paniculata* leaf essential oil. SD: Standard deviation. Values represent the mean ± SD of four replicates.

Furthermore, probit analysis confirmed the larvicidal activity of the oil, yielding a median lethal concentration ($LC_{50}$) of 61.56μg/mL (95% CI: 22.23−140.78μg/mL). These toxicity parameters and statistical fit indicators ($\chi^{2}=0.479$, DF=1) are summarized in Table 6. According to established larvicidal classification frameworks [43, 44], natural extracts presenting $LC_{50}$ values between $50100\;\mathrm{and}\;\;\mu g\;{\mathrm{mL}}^{-1}$ are categorized as moderately active, demonstrating promising larvicidal potential against mosquito larvae. An $LC_{50}$ value below 100μg/mL underscores the strong bioactivity of the crude leaf essential oil against *A. aegypti*, which may be attributed to the predominance of active sesquiterpene constituents. However, given that this evaluation relied on three concentration levels as a preliminary screening, and considering the wide confidence interval (CI) obtained, these results should be interpreted cautiously as an initial concentration‐response assessment rather than a precise determination of larvicidal potency. Full‐range WHO‐standardized protocols utilizing 5 to 7 narrower concentration steps will be required in subsequent studies to reliably refine these toxicological parameters without statistical extrapolation.

**TABLE 6:** Lethal concentration ($LC_{50}$) of *E. paniculata* leaf essential oil against *A. aegypti* larvae.

Sample	LC_50_ (µg/mL) (LCL—UCL)	*x* ^2^	DF
EpEO	61.56 (22.23 – 140.78)	0.479	1^*^

$LC_{50}$: Lethal concentration required to kill 50% of larvae. LCL: Lower confidence limit (95%). UCL: Upper confidence limit (95%). $\chi^{2}$: Chi‐square value. DF: Degrees of freedom.

* *p* > 0.05 indicates no significant difference between observed and expected frequencies.

In our assays, the essential oil of *E. paniculata* exhibited an $LC_{50}$ of 61.56μg/mL, demonstrating natural larvicidal activity. Although synthetic larvicides such as temephos exhibit significantly lower $LC_{50}$ values in susceptible *A. aegypti* strains (typically in the μg/L range) [45], temephos served in this study as an external literature reference benchmark rather than a concurrent experimental control. The results highlight the potential of plant‐based extracts as complementary candidates in integrated vector management. While botanical larvicides are often hypothesized to possess lower environmental persistence and distinct modes of action compared to conventional synthetics, comprehensive ecotoxicological assays against nontarget aquatic organisms and resistance‐selection studies are required to confirm these potential benefits for *E. paniculata* essential oil. Furthermore, directly comparing crude essential oil potency to pure monomolecular larvicides must account for the complex mixture of components in natural extracts; future standardized trials will include concurrent commercial positive controls under identical experimental setups.

These results suggest that the active compounds present in the essential oil of *E. paniculata* exhibit larvicidal potential, which may involve AChE inhibition as one plausible contributing neurotoxic mechanism among potential multi‐target interactions typical of essential oils. By inhibiting AChE, the essential oil prevents the breakdown of acetylcholine at synaptic junctions, leading to the accumulation of this neurotransmitter [46]. This excessive stimulation of cholinergic receptors disrupts normal nerve impulse transmission, causing continuous muscle contraction and paralysis in mosquito larvae. Furthermore, at higher concentrations, plant‐derived compounds may also compromise the histological integrity of larvae, leading to mortality [47]. Such mechanisms are frequently reported for plant‐derived neurotoxic compounds and support the potential of *E. paniculata* as a natural larvicide.

Inhibition of AChE is a mechanism frequently associated with toxicity in insects, as it interferes with the transmission of nerve impulses, leading to paralysis and death of the larva. In this respect, essential oils can act as selective neurotoxic agents, proving to be a potential alternative for the biological control of arbovirus vectors. Furthermore, studies have already attributed the presence of sesquiterpenes as bioactive compounds with larvicidal potential [48, 49]. The high concentration of these compounds in the dried leaf sample may be directly related to the observed effects, reinforcing the hypothesis that this class of organic compounds plays a central role in the bioactivity of essential oils.

No toxicity studies against *A. aegypti* were found with species of the *Endlicheria* genus, but publications with Lauraceae species are promising. Favorable results were found for the *Aniba duckei* species which showed larvicidal activity of the volatile oil, the authors highlighted its potential use in larvicidal formulations for domestic use [50]. The species *Aniba rosaeodora* Ducke also showed larvicidal activity with a LC50 of 41.07 mg L‐1. According to the authors of this analysis, it was possible to assess that the essential oil of the species is composed of substances that have a larvicidal effect against *A. aegypti*. The majority constituents identified in this study were *β*‐linalool (63.15%), linalool transfuran oxide (9.73%), and linalool cis‐furan oxide (7.69%) [51]. In another study, the essential oil from the leaves of the species *Ocotea nutans* showed remarkable potential for larvicidal activity with a LC50 of 250 µg/mL [52]. Furthermore, the essential oil from the leaves of *Neolitsea ellipsoidea*, rich in (E)‐*β*‐ocimene (87.6%), showed excellent larvicidal activity against *A. aegypti* with LC50 of 6.59 µg/mL [53]. n comparison with other Lauraceae species, the major constituents of *E. paniculata* essential oil differ notably. While *Aniba rosaeodora* oil is rich in β‐linalool and its derivatives [51], and *Neolitsea ellipsoidea* is dominated by (E)‐β‐ocimene [53], *E. paniculata* essential oil contains a higher proportion of sesquiterpenes, which may contribute to its unique larvicidal profile. These differences in chemical composition among Lauraceae species may underlie variations in larvicidal potency and mechanisms of action.

These findings highlight species of the Lauraceae family as potential larvicidal agents, containing various volatile compounds that can interfere with the development and life cycle of mosquito larvae, particularly sesquiterpenes and monoterpenes, which are the main components of essential oils. No previous toxicity studies against *A. aegypti* have been conducted with essential oils from *Endlicheria* species, which emphasizes the novelty of our findings. With an ${\mathrm{LC}}_{50}=61.56\;\mu g/\mathrm{mL}$, *E. paniculata* oil demonstrates promising larvicidal potential against *A. aegypti*. This effect may involve a chemical interaction between *amorpha‐4,9‐dien‐2‐ol* and the sesquiterpene matrix, which could theoretically hinder the rapid onset of resistance compared to monomolecular active ingredients; however, this remains a hypothesis that warrants dedicated long‐term selection and resistance monitoring studies.

### 2.4 Molecular Docking

#### 2.4.1 Target Quality Analysis

The quality of the generated model was estimated using Global Model Quality Estimation (GMQE), expressed as a number between 0 and 1, where values close to 1 reflect higher accuracy and reliability of the constructed 3D protein model [54]. The GMQE for the model was 0.83, with a sequence identity of 92.58%, which validates the accuracy and reliability of the generated model. Furthermore, the Ramachandran plot of the model showed 95.14% of the residues within the most favorable regions, indicating that the AChE from *Ae. aegypti* represents a reliable model (Figure 2). This is corroborated by the fact that an occupancy value higher than 90% in the most favorable region indicates a reliable PDB model for pharmacodynamic simulations [55]. Local per‐residue quality estimates further confirmed high geometric accuracy throughout the active site gorge and peripheral anionic site (PAS).

**FIGURE 2:**
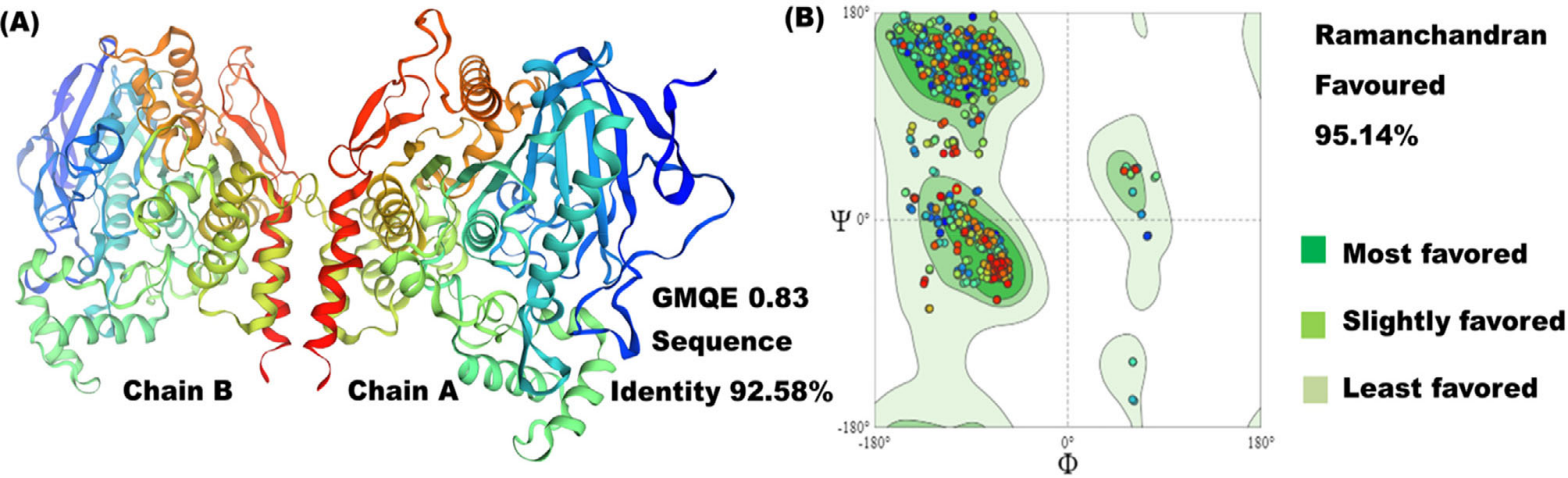
Structural validation and conformational analysis of AChE from *Ae. Aegypti*. (A), shows the AChE structure. (B), presents the Ramachandran plot for the corresponding protein, indicating that most residues are located in favorable or allowed regions, corroborating the structural quality of the model used in the molecular docking simulations.

#### 2.4.2 Molecular Docking Simulations

Seeking alternatives to synthetic insecticides, the functional AChE‐1 (AChE‐1, UniProt Q6A2E2) from *Ae. aegypti* was used as a target in molecular docking assays to investigate the potential larvicidal mechanism of the major constituents present in the essential oil of *E. paniculata*. To provide a comprehensive computational screening of the species, the docking analysis evaluated the major volatile constituents representative of leaf, bark, and wood essential oils. Chemical traceability, PubChem database identifiers, SMILES strings, and protonation states of all evaluated constituents are provided in Table S2, while target protein parameters and grid box dimensions are listed in Table S3. Docking protocols were validated via re‐docking of the co‐crystallized inhibitor from the template structure (PDB ID: 6ARY), yielding a heavy‐atom RMSD of 1.18Å (<2.0Å) and reproducing key active site interactions. To benchmark the vina docking score scores of the volatile constituents, standard AChE inhibitors (neostigmine and temephos‐oxon) were evaluated under identical parameters as positive controls, displaying reference binding energies of −7.1kcal and $-7.8\;\mathrm{kcal}\cdot \mathrm{mo}l^{-1}$, respectively. Following the in silico trials, conformational clustering RMSD values across independent runs ranged between 1.148 and 1.864 Å (confirming pose convergence below the ≤2.0Å threshold), and affinity energies ranged from −7.4 to $-5.2\;\mathrm{kcal}\cdot \mathrm{mo}l^{-1}$ (Table 7). These values demonstrate that the major constituents bind within a comparable energy range to established cholinesterase inhibitors. Regarding the binding energy, the data showed that, with the exception of tetradecanal, all compounds displayed favorable vina docking score (equal to or lower than $-6.0\;\mathrm{kcal}\cdot \mathrm{mo}l^{-1}$) [56]. Among these, viridiflorol and selina‐3,7(11)‐diene stood out, exhibiting the most significant docking score values ($-7.4\;\mathrm{kcal}\cdot \mathrm{mo}l^{-1}$). While viridiflorol highlights the binding potential of bark‐derived metabolites, the bioactivity observed experimentally for the leaf oil is attributed to its specific profile, including major constituents such as amorpha‐4,9‐dien‐2‐ol ($-7.0\;\mathrm{kcal}\cdot \mathrm{mo}l^{-1}$). Interaction analysis revealed that the evaluated compounds predominantly established hydrophobic interactions. In addition to these interactions, hydrogen bonds were also observed in the complexes formed between the AChE from *Ae. aegypti* and the compounds *β*‐muurola‐4,10(14)‐dien‐1‐ol, viridiflorol, and amorpha‐4,9‐dien‐2‐ol (Table 7). The top‐ranked docking poses were mapped directly relative to key functional sub‐sites of the active gorge: the catalytic triad (Ser200, His440, Glu327), the acyl‐binding pocket (Phe295, Phe297), and the peripheral anionic site (PAS; Trp286, Tyr124, Tyr341). Poses generated within Chain A and Chain B of the homodimeric template reflect structurally equivalent binding microenvironments within identical, symmetrical catalytic gorges. Overall, the in silico molecular docking simulations provide theoretical insights into potential ligand‐protein interactions, supporting AChE inhibition as a plausible contributing hypothesis rather than a definitive sole mechanism of action.

**TABLE 7:** Docking score, RMSD, and types of interactions formed by the major constituents of the essential oil from *E. paniculata* against AChE‐1 from *Ae. aegypti*.

Major constituent	Docking score (kcal/mol)	RMSD (Å)	Interaction type	Residue (Distance in Å)
β‐muurola‐4,10(14)‐dien‐1‐ol	−6.8	1.649	Hydrophobic	Trp408A (3.68), Trp408A (3.68), Leu411A (3.71), Phe416A (3.84), Phe457A (3.30), Tyr460A (3.49), Tyr461A (3.72)
			Hydrogen Bond	Cys414A (2.96)
δ‐elemene	−6.7	1.148	Hydrophobic	Trp408B (3.72), Trp408B (3.49), Leu411B (3.96), Phe416B (3.55), Tyr456B (3.77), Phe457B (3.76), Phe457B (3.24), Tyr460B (3.66), Tyr461B (3.53)
Tetradecanal	−5.2	1.799	Hydrophobic	Trp212B (3.58), Phe416B (3.77), Tyr456B (3.28), Phe457B (3.64), Phe457B (3.80), Tyr460B (3.60), Tyr460B (3.62)
Isobornyl acetate	−6.6	1.515	Hydrophobic	Trp408A (3.59), Trp408A (3.81), Phe416A (3.57), Tyr456A (3.70), Tyr460A (3.90), Tyr461A (3.51)
Spathulenol	−7.0	1.450	Hydrophobic	Phe416A (3.37), Tyr456A (3.74), Phe457A (3.72), Tyr460A (3.56), Tyr460A (3.40), Tyr460A (3.71), Tyr461A (3.22)
Viridiflorol	−7.4	1.680	Hydrophobic	Ile413B (3.79), Phe416B (3.88), Tyr456B (3.76), Phe457B (3.35), Tyr460B (3.68), Tyr460B (3.28)
			Weak Polar/Electrostatic	Tyr460B (3.65)
Amorpha‐4,9‐dien‐2‐ol	−7.0	1.846	Hydrophobic	Tyr249B (3.89), Trp408B (3.91), Trp408B (3.59), Trp408B (3.77), Ile413B (3.93), Phe457B (3.70)
			Hydrogen Bond	Glu415B (3.10)
Selina‐3,7(11)‐diene	−7.4	1.498	Hydrophobic	Trp408A (3.77), Trp408A (3.72), Trp408A (3.63), Tyr456A (3.45), Phe457A (3.28), Tyr460A (3.50), Tyr460A (3.87), Tyr461A (3.71), Tyr461A (3.62)
γ‐elemene	−6.6	1.864	Hydrophobic	Trp408A (3.70), Trp408A (3.71), Trp408A (3.66), Leu411A (3.71), Tyr461A (3.69), Tyr461A (3.49)

Figure 3 highlights the binding modes of the compounds that exhibited the most significant docking score values: viridiflorol and selina‐3,7(11)‐diene ($-7.4\;\mathrm{kcal}\cdot \mathrm{mo}l^{-1}$). Both ligands accommodate within the internal catalytic gorge of AChE‐1 in close proximity to the catalytic Ser200 residue, maintaining minimum atomic distances of 3.4Å (viridiflorol) and 3.8Å (selina‐3,7(11)‐diene), thereby physically occluding access to the catalytic triad.

**FIGURE 3:**
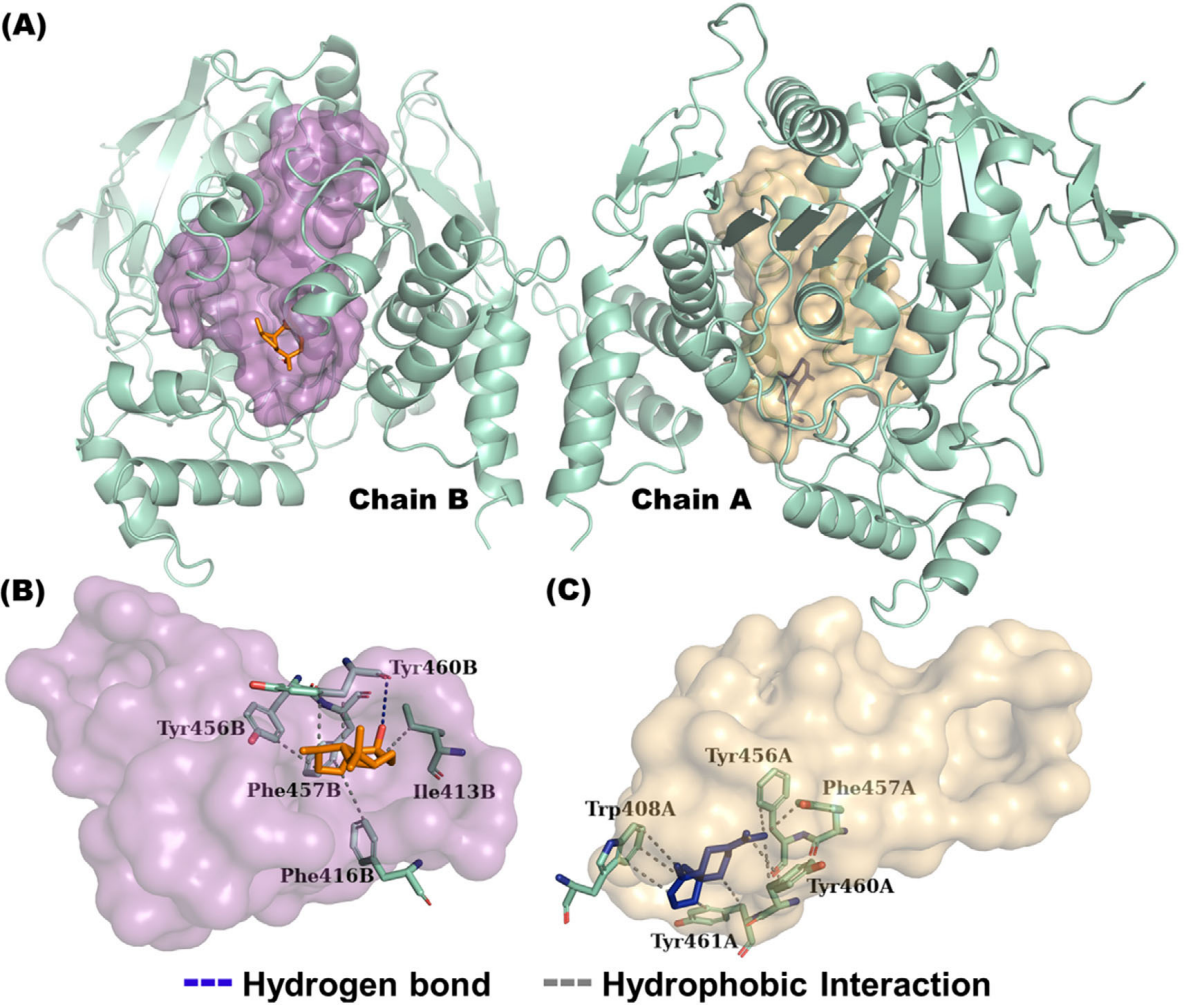
Structural mapping of ligand binding modes within the AChE‐1 active site gorge of *Ae. aegypti*. (A) Overview of AChE‐1 complexed with major essential oil constituents positioned within the catalytic gorge. (B) Detailed binding interaction pocket of viridiflorol, highlighting proximity to catalytic Ser200 (3.4Å), hydrophobic contacts, and weak polar interaction with Tyr460B (3.65Å). (C) Detailed binding interaction pocket of selina‐3,7(11)‐diene (3.8Å from Ser200), showing hydrophobic stabilization within the acyl‐binding and aromatic sub‐sites.

It is possible to observe that the AChE‐1/viridiflorol complex showed a predominance of hydrophobic interactions with the nonpolar side‐chain residue Ile413B (3.79Å) and aromatic residues Phe416B (3.88Å), Tyr456B (3.76Å), Phe457B (3.35Å), and Tyr460B (3.28 and 3.68Å), in addition to a weak polar/electrostatic contact [57] with the aromatic residue Tyr460B (3.65Å) (Figure 3B). On the other hand, the AChE‐1/selina‐3,7(11)‐diene complex exhibited hydrophobic interactions with the aromatic residues Trp408A (3.63, 3.72, and 3.77Å), Tyr456A (3.45Å), Phe457A (3.28Å), Tyr460A (3.50 and 3.87Å), and Tyr461A (3.62 and 3.71Å) (Figure 3C).

## 3 Conclusions

In conclusion, this study provides the first comprehensive characterization of the volatile profiles of *E. paniculata*, revealing a distinct tissue‐specific distribution of sesquiterpenoids. While the wood essential oil is dominated by β‐muurola‐4,10(14)‐dien‐1‐ol and the bark oil by spathulenol, the leaf oil is uniquely characterized by the rare oxygenated sesquiterpene amorpha‐4,9‐dien‐2‐ol as its primary constituent. This finding represents a significant chemotaxonomic marker for the genus, as the occurrence of this compound as a major component is scarcely reported in the literature.

Additionally, the leaf essential oil demonstrated larvicidal activity under laboratory conditions against *A. aegypti*. Regarding the enzymatic assays, the oil showed preliminary in vitro inhibitory potential against AChE and α‐glucosidase (with inhibition rates exceeding 97% at single screening concentrations). These preliminary observations provide a foundation for future studies to determine detailed inhibition kinetics ($IC_{50}$ and $K_{i}$) and identify the specific active metabolites through bio‐guided isolation.

Complementing these experimental findings, molecular docking simulations inferred that, with the exception of tetradecanal, the major constituents of the essential oil warrant further evaluation as prospective candidates for vector control research. These compounds displayed favorable predicted binding energy scores towards *Ae. aegypti* AChE‐1, highlighted by the receptor–ligand complexes formed by viridiflorol and selina‐3,7(11)‐diene, which achieved the highest predicted affinity scores ($-7.4\;\mathrm{kcal}\cdot \mathrm{mo}l^{-1}$, comparable to the commercial reference controls neostigmine and temephos‐oxon). The predicted ina docking score of these complexes was predominantly driven by hydrophobic interactions and proximity to the catalytic triad (Ser200), as well as specific polar contacts. These in silico data support AChE‐1 active gorge occlusion as a plausible structural hypothesis contributing to the observed bioactivity.

Ultimately, our findings position *E. paniculata* as a relevant plant species for further investigation into bioactive natural compounds, providing an original and multi‐disciplinary contribution to the phytochemistry of the Lauraceae family.

## 4 Experimental Section

### 4.1 Plant Material Collection and Identification

Samples of *E. paniculata* (leaves, bark, and wood) were collected in April 2023 at the Saint‐Hilaire‐Lange National Park, Municipality of Matinhos, State of Paraná, Brazil. The collection sites were located at coordinates 25.7902°S, 48.5618°W; 25.7880°S, 48.5634°W; and 25.7880°S, 48.5622°W. Botanical identification was performed by Wanderlei do Amaral. Samples of the species were deposited in the Herbarium of the Municipal Botanical Museum (MBM) in Curitiba, Paraná‐BR and registered under voucher number MBM46834. This research was authorized by the National System for the Management of Genetic Heritage and Associated Traditional Knowledge (SisGen) under No. AFB5575.

### 4.2 Essential Oil Extraction and Yields

The extraction of essential oils (EOs) from the bark and wood was performed at the Laboratory of Natural Products of the Federal University of Paraná (Setor Litoral), Matinhos, PR, Brazil. For each tissue, 100 g of air‐dried material was subjected to hydrodistillation in a 2000 mL round‐bottom flask containing 1000 mL of distilled water. The process was conducted in triplicate using a Clevenger‐type apparatus for 6 h [58]. The temperature was initially maintained at maximum until boiling and then adjusted using heating mantles to ensure a constant reflux. The obtained EOs were stored in a freezer (‐4°C) in amber glass vials to prevent volatilization.

The extraction of the leaf EO was carried out at the Department of Pharmacy of the Federal University of Paraná, Curitiba, PR, Brazil. The leaves were dried at room temperature (protected from light and moisture) for 15 days, then ground and subjected to hydrodistillation for 6 h [58]. A total of 603 g of dried material was processed in a 3000 mL flask with a sufficient amount of distilled water. The leaf EO was stored under the same conditions described above to prevent chemical degradation.

The EO yields for all tissues were calculated on a volume/weight (v/w) basis, expressed as mL of oil per 100 g of dried plant material [58]. Due to low extraction yields, the bark and wood EOs were not subjected to enzyme inhibition and larvicidal assays.

### 4.3 Chemical Analysis of Essential Oils

The chemical composition of the essential oils was determined using gas chromatography coupled to a mass spectrometer (GC/MS) and gas chromatography equipped with a flame ionization detector (GC‐FID). The essential oils were diluted in 1% dichloromethane (v/v) to reduce viscosity and enhance volatility.

GC‐MS analyses were performed on a Shimadzu‐2010 Plus mass spectrometer detector coupled to a gas chromatograph. A 1.0 µL volume of the solution was injected at a split ratio of 1:20. The injector was kept at 250°C. The separation of the constituents was achieved on a HP‐5MS capillary column (30 m × 0.25 mm × 0.25 µm) using helium as the carrier gas at a constant flow rate of 1.0 mL min^−^^1^. The oven temperature was programmed from 60 to 240°C at a rate of 3°C min^−^^1^. The mass detector was operated in electron ionization mode (70 eV) at a scan rate of 3.15 scans s^−^^1^ and a mass range of 40 to 450 *m/z*. The transfer line was kept at 260°C, the ion source at 230°C, and the analyzer (quadrupole) at 150°C.

For quantification, the same diluted samples were injected into an Agilent 7890A gas chromatograph equipped with a flame ionization detector (FID) operated at 280°C. The GC‐FID analysis was performed using the same HP‐5MS column and temperature program described for GC‐MS, but utilizing hydrogen as the carrier gas at a flow rate of 1.5 mL min^−^^1^. The percentage composition was obtained by electronically integrating the FID signal. The relative area percentage for each constituent was calculated as the average of three independent injections, and results are expressed as mean ± standard deviation (SD). All analyses were performed in triplicate to ensure analytical precision, reproducibility, and acceptable error margins.

The chemical constituents were identified by comparing their mass spectra and linear retention indices (LRI) with experimental data reported by Adams [59]. The LRI values were determined from the injection of a homologous series of *n*‐alkanes (C_7–_C_30_, Sigma‐Aldrich, St. Louis, MO, USA) under the same chromatographic conditions, using the Van den Dool and Kratz equation for temperature‐programmed gas chromatography [60]. Compound identification confidence and reporting parameters were aligned with the Metabolomics Standards Initiative (MSI) guidelines. Constituents were evaluated based on their chromatographic retention time ($R_{t}$), calculated linear retention index ($RI_{\mathrm{calc}}$), literature retention index ($RI_{\mathrm{lit}}$), and index deviation ($\Delta \mathrm{RI}\;=RI_{\mathrm{calc}}\;-RI_{\mathrm{lit}}$). A maximum allowable deviation window of ΔRI≤±35 units was accepted, which accounts for minor variations in column film thickness, heating rate, and stationary phase interactions typical for complex sesquiterpene skeletons. Mass spectral matching against the Adams (2017) library required both forward similarity and reverse‐match scores above 90%, together with the verification of key diagnostic m/z ions for structural confirmation. According to the MSI criteria, all identified constituents represent Level 2 (Tentatively Identified Compounds), as identification was based solely on EI mass‐spectrum and retention index matching without confirmation by commercial authentic standards (Level 1). Furthermore, due to the nonchiral nature of the GC‐MS analysis, chemical structures represent tentative assignments based on MS and retention index matches, particularly where stereoisomers or closely related sesquiterpene skeletons cannot be distinguished unequivocally without absolute stereochemical determination. The total ion chromatograms (TIC) of the essential oils from wood, bark, and leaves, as well as the *n*‐alkane series used for retention index calculations, are provided in the Supporting Information (Figures S1–S4). Additionally, the mass spectra of the major identified constituents are presented in Figures S5–S22.

### 4.4 Enzyme Inhibition Assays

Enzyme inhibition tests with essential oil from *E. paniculata* leaves were carried out at the Natural Products Research Laboratory of the Department of Pharmaceutical Sciences at the Regional University of Blumenau (FURB). All enzyme inhibition evaluations were performed as a preliminary single‐concentration screening. Assays were conducted across three independent biological experiments (n=3), with each experiment evaluated in technical triplicate.

### 4.5 *α*‐Glucosidase Inhibition Assay

The α‐glucosidase inhibition assay was performed as described by Kim et al. [61], with minor modifications to establish exact final well concentrations. Solutions of *E. paniculata* leaf essential oil were prepared in methanol at a stock concentration of $1\;\mathrm{mg}\;mL^{-1}$ and diluted with potassium phosphate buffer (pH6.8, $0.1\;\mathrm{mol}\;L^{-1}$) to a pre‐addition concentration of $500\;\mu g\;{\mathrm{mL}}^{-1}$. An aliquot of 50μL of α‐glucosidase solution ($1\;U\;mL^{-1}$, Type I from *Saccharomyces cerevisiae*, Sigma‐Aldrich, Cat. No. G5003) was premixed with 20μL of the sample solution ($500\;\mu g\;{\mathrm{mL}}^{-1}$) and 570μL of potassium phosphate buffer (pH6.8, $0.1\;\mathrm{mol}\;L^{-1}$). All tubes were vortexed and incubated in a water bath at 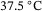 for 20min. Afterwards, 100μL of p‐nitrophenyl‐α‐D‐glucopyranoside (pNPG, $1\;\mathrm{mmol}\;L^{-1}$) was added as substrate to initiate the reaction. The tubes were shaken again and incubated for 30min in a water bath at 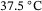, followed by the addition of 650μL of $1\;M\;Na_{2}CO_{3}$ solution to terminate the enzymatic activity (total reaction mixture volume = 1390μL, resulting in a final nominal assay concentration of $7.19\;\mu g\;{\mathrm{mL}}^{-1}$ for the essential oil and a final solvent concentration of <1.5%v/v methanol). The amount of p‐nitrophenol formed was measured in a spectrophotometer at λ=410nm. Acarbose ($50\;\mu g\;{\mathrm{mL}}^{-1}$ pre‐addition) was used as a reference positive control. A negative control containing the solvent vehicle without essential oil was prepared under identical conditions. For each sample, an individual sample blank (containing 20μL of sample solution, buffer, and substrate without enzyme) was evaluated to subtract any intrinsic optical background or turbidity. The percentage of enzyme inhibition was calculated using Equation 1:

$$\begin{array}{ccc} & & \mathrm{Inhibitory}\;\mathrm{Activity}\;(\%)\;\\ & = & \frac{\mathrm{Ab}s_{\mathrm{Negative}\;\mathrm{Control}}-\left(\mathrm{Ab}s_{\mathrm{Sample}}-\mathrm{Ab}s_{\mathrm{Sample}\;\mathrm{Blank}}\right)}{\mathrm{Ab}s_{\mathrm{Negative}\;\mathrm{Control}}}\;\times 100 \end{array}$$

### 4.6 Acetylcholinesterase (AChE) Inhibition Assay

Acetylcholinesterase (AChE) inhibitory activity was evaluated in vitro using commercial AChE from *Electrophorus electricus* (Sigma‐Aldrich) according to the colorimetric method described by Ellman et al. [62], adapted for preliminary single‐concentration screening. Essential oil samples were diluted in methanol at a stock concentration of $1\;\mathrm{mg}\;mL^{-1}$. In each test tube, 325μL of Tris‐HCl buffer ($50\;\mathrm{mmol}\;L^{-1}$, pH8.0) was added. An aliquot of 100μL of essential oil solution ($1\;\mathrm{mg}\;mL^{-1}$) and 20μL of AChE solution ($0.25\;U\;mL^{-1}$, Type VI‐S from *Electrophorus electricus*, Sigma‐Aldrich, Cat. No. C3389, diluted in Tris‐HCl buffer containing 0.1%w/v Bovine Serum Albumin) were added, and the mixture was incubated at room temperature for 15min. Then, 70μL of acetylthiocholine iodide (ATCI, $0.021\;\mathrm{mg}\;mL^{-1}$) substrate solution and 470μL of Ellman's reagent ($5,5^{\prime }$‐dithiobis(2‐nitrobenzoic acid), DTNB, prepared in Tris‐HCl buffer supplemented with 1MNaCl and $0.02\;M\;\mathrm{MgC}l_{2}\cdot 6H_{2}O$) were added. After homogenization, the tubes were incubated in the dark for 25min. Subsequently, 1000μL of Tris‐HCl buffer was added (total reaction mixture volume = 1985μL, yielding a final nominal assay concentration of $50.38\;\mu g\;{\mathrm{mL}}^{-1}$ for the essential oil and a final methanol concentration of 5.0%v/v). The absorbance of the formed yellow 5‐thio‐2‐nitrobenzoate anion was measured at λ=405nm. Neostigmine hydrochloride ($100\;\mu g\;{\mathrm{mL}}^{-1}$ stock, final assay concentration $5.04\;\mu g\;{\mathrm{mL}}^{-1}$) was used as a reference positive control, and the solvent vehicle served as the negative control. Sample blanks containing essential oil, DTNB, and buffer without enzyme were read to correct for background interference. The inhibitory activity was calculated using Equation 2:

$$\mathrm{Inhibitory}\;\mathrm{Activity}\;(\%)\;=\left[1-\frac{\mathrm{Ab}s_{\mathrm{Sample}}-\mathrm{Ab}s_{\mathrm{Sample}\;\mathrm{Blank}}}{\mathrm{Mean}\;\mathrm{Ab}s_{\mathrm{Negative}\;\mathrm{Control}}}\right]\;\times 100$$

### 4.7 Statistical Analysis

All enzyme inhibition experiments were performed in three independent biological repetitions (n=3), with each biological replicate assayed in technical triplicate. Results are expressed as mean ± standard deviation (SD). Prior to parametric statistical testing, data normality and homogeneity of variances were confirmed using the Shapiro‐Wilk and Levene tests, respectively. Significant differences among sample groups and reference controls were analyzed by one‐way analysis of variance (ANOVA) followed by Tukey's post‐hoc test for multiple comparisons, with two‐sided significance set at p<0.05(∗p<0.05, p<0.01, ∗∗∗p<0.001). Statistical procedures and graphs were generated using GraphPad Prism software version 8.0 (GraphPad Software, San Diego, CA, USA).

### 4.8 Larvicidal Activity Assay

Larvicidal activity assay with the essential oil from *E. paniculata* leaves against *A. aegypti* larvae were carried out at the Pharmacotechnical Laboratory of the Pharmacy Department of the Federal University of Paraná (UFPR).

The methodology used was adapted to that of Garcez et al. and Betim et al. [63, 52]. The Rockefeller strain used was provided by the Oswaldo Cruz Foundation. To evaluate the activity of *E. paniculata* essential oil, *A. aegypti* eggs were hatched in the laboratory at (25 ± 3°C), in a relative humidity of 80% (BOD greenhouse) and fed with fish feed (Alcon basic, MEP 200 complex) up to the third stage of larval development.

The essential oil samples were emulsified using 0.5% DMSO (dimethylsulfoxide) to prepare test solutions at concentrations of 10, 100, and 1000 µg/mL as a preliminary concentration‐response screening. Ten larvae were added per beaker and exposed for 24 h to the test solutions, negative control (distilled water), or solvent control (0.5% DMSO). After the exposure period, live and dead larvae from each treatment were counted. All assays were performed in quadruplicate (n=4), evaluating a total of 40 larvae per concentration.

### 4.9 Statistical Analysis

The larvicidal assay was carried out in quadruplicate (n=4), 40 larvae per concentration), and mortality data are presented as absolute counts and percentages. Data normality and model assumptions were evaluated prior to analysis, and no outliers were excluded. Mortality data were subjected to Probit analysis to calculate the median lethal concentration ($LC_{50}$) with its 95% CI, and model goodness‐of‐fit was assessed using the Pearson Chi‐square test ($\chi^{2}$). Statistical analyses were performed using IBM SPSS Statistics version 20.0 (IBM Corp., Armonk, NY, USA).

### 4.10 Molecular Docking Studies

#### 4.10.1 Ligand and Target Preparation

The chemical structures of the major constituents were rendered, and their lowest energy conformers were saved at physiological pH using MarvinSketch software (v. Chemaxon). Geometry optimization was subsequently performed using Avogadro software, configured with the steepest descent algorithm and applying the MMFF94 (Merck Molecular Force Field) force field [64]. Docking simulations were performed using the predominant natural stereoisomers reported in the literature for Lauraceae species. However, as nonchiral GC‐MS does not establish absolute configuration, these assignments remain tentative.

To investigate the mechanism of action of these volatile constituents against *A. aegypti*, the amino acid sequence of acetylcholinesterase‐1 (AChE‐1) was retrieved from the Universal Protein Resource (UniProt) database under the accession identifier Q6A2E2 [65]. AChE‐1 was selected as the target model because it is the primary functional synaptic cholinesterase in the central nervous system of insects and the major target associated with neurotoxicity and resistance in mosquitoes. Homology modeling of the protein target was carried out via the Swiss‐Model server, configured to search for structural templates through alignment tests using the HMM‐HMM‐based lightning‐fast iterative sequence search (HHBlits) algorithm [66, 67]. Following sequence alignment, the crystal structure of an insecticide‐resistant AChE mutant from the malaria vector *Anopheles gambiae* (PDB ID: 6ARY) was selected as the template to model the AChE of *Ae. aegypti* [66, 68]. The target receptor was constructed as a functional homodimer consisting of Chains A vina docking score and B. The modeled sequence was explicitly verified to contain the wild‐type Glycine residue at position 119 (Gly119), correcting the G119S mutation present in the template to accurately reflect the wild‐type susceptible phenotype. For target preparation prior to the molecular docking simulations, polar hydrogens and Kollman charges were added, and Gasteiger charges were calculated [69] utilizing AutoDockTools software [70]. To ensure that the inhibitor‐bound template conformation did not constrain the binding site, active site side‐chain geometries were relaxed prior to docking.

### 4.11 Model Validation and Structural Quality

Validation and structural quality control of the constructed AChE model were performed through Global Model Quality Estimation (GMQE) analysis, QMEANDisCo global scoring (0.78±0.05), and Ramachandran plots generated by the Swiss‐Model server. The GMQE index was used to estimate the overall quality of the modeled structure, while the Ramachandran plot estimated the percentage of amino acid residues situated within stereochemically favorable regions. Local per‐residue quality estimates confirmed high accuracy throughout the catalytic gorge and PAS. The surface area of the binding pocket was predicted using the ProteinsPlus web server, and the cavity surface plot was employed to identify potential binding sites.

### 4.12 Molecular Docking Simulation and Data Output

Molecular docking simulations were performed using AutoDock Vina (v. 1.2.0) [71], employing an iterated local search global optimizer (Monte Carlo sampling) with an exhaustiveness setting of 64 [72] and the default Vina scoring function. The search space was centered on the catalytic gorge of AChE‐1 with center coordinates set at x=−70.418, y=49.463, and z=41.364, with search box dimensions of 41.25×44.25×47.25Å (corresponding to 110×118×126 grid dimensions mapped at 0.375Å grid spacing). This grid size encompassed the entire active site gorge and PAS while maintaining focused search reliability for ligand binding modes. A total of 50 independent runs were performed, generating 20 poses per simulation. To validate the docking protocol, re‐docking of the co‐crystallized inhibitor from the template structure (PDB ID: 6ARY) into the AChE‐1 active site gorge was performed, yielding a heavy‐atom RMSD of 1.18Å (<2.0Å threshold) and reproducing the key catalytic site interactions. To contextualize the Vina docking scores of the volatile constituents, standard AChE inhibitors (neostigmine and temephos‐oxon) were evaluated under identical parameters as positive benchmark controls, displaying reference Vina docking scores of −8.2kcal/mol and −7.9kcal/mol, respectively. For the tested volatile constituents, an RMSD criterion of <2.0Å [73] was applied as a conformational clustering threshold to confirm pose reproducibility across independent simulation runs, with the top‐ranked pose selected based on the lowest predicted Vina docking score [56].

### 4.13 Visualization of Binding Modes and Receptor–Ligand Interactions

The docking results were analyzed using UCSF Chimera [74], PyMOL [75], and Discovery Studio Visualizer viewer [76] softwares. To identify and visualize specific molecular interactions and hydrogen bonds, the web‐based Protein‐Ligand Interaction Profiler (PLIP) server was employed [77, 78].

## Author Contributions

**Jenifer Priscila de Araujo**: project administration, conceptualization, methodology, software, data curation, investigation, validation, formal analysis, visualization, writing – original draft, writing – review and editing. **Luiz Everson Da Silva**: conceptualization, investigation, resources, writing – review and editing. **Wanderlei do Amaral**: conceptualization, investigation, writing – review and editing. **Michele Debiasi Alberton**: methodology, investigation, validation, formal analysis, resources, writing – review and editing. **Ana Helena Loos Moritz**: methodology, investigation, formal analysis, writing – review and editing. **Fernando Cesar Martins Betim**: methodology, investigation, writing – review and editing. **Deise Prehs Montrucchio**: conceptualization, writing – review and editing. **Marilis Dallarmi Miguel**: conceptualization, writing – review and editing. **Josiane de Fátima Gaspari Dias**: conceptualization, methodology, software, investigation, validation, formal analysis, resources, writing – review and editing. **Hélcio Silva Dos Santos**: methodology, software, investigation, validation, formal analysis, visualization, resources, writing – review and editing. **Emmanuel Silva Marinho**: methodology, software, investigation, formal analysis, visualization, writing – review and editing. **Márcia Machado Marinho**: methodology, software, investigation, formal analysis, visualization, writing – review and editing. **Jacilene Silva**: methodology, software, investigation, formal analysis, visualization, writing – review and editing. **Obdulio Gomes Miguel**: supervision, conceptualization, methodology, investigation, validation, resources, writing – review and editing.

## Conflicts of Interest

The authors declare no conflicts of interest.

## Supporting information




**Supporting File 1**: Cbdv71881‐sup‐0001‐SuppMat.pdf.

## Data Availability

The data that supports the findings of this study are available in the Supporting Information of this article.
