# Supplementary material for: α‐Glucosidase and Acetylcholinesterase (AChE) Inhibitory Activities, Larvicidal Potential and GC–MS Analysis of Endlicheria paniculata: A Combined Experimental and Molecular Docking Study
Source: Chem Biodivers. 2026 Oct 27;23(10):e71881. doi: 10.1002/cbdv.71881 (PMC13711495; doi:10.1002/cbdv.71881)
Supplement: Supplementary file 1 [file CBDV-23-e71881-s001.pdf]

SUPPLEMENTARY MATERIAL

α-Glucosidase and Acetylcholinesterase (AChE) Inhibitory Activities, Larvicidal Potential and GC–MS Analysis of *Endlicheria paniculata*: A Combined Experimental and Molecular Docking Study

Jenifer Priscila de Araujo[a*], Luiz Everson da Silva[b], Wanderlei do Amaral[c], Michele Debiasi Alberton[d], Ana Helena Loos Moritz[d], Fernando Cesar Martins Betim[a], Deise Prehs Montrucchio[a], Marilis Dallarmi Miguel[a], Josiane de Fátima Gaspari Dias[a], Hélcio Silva dos Santos[e], Emmanuel Silva Marinho[e], Márcia Machado Marinho[e], Jacilene Silva[e] and Obdulio Gomes Miguel[a]

[a] Federal University of Paraná, Postgraduate Program in Pharmaceutical Sciences, Department of Pharmacy, Curitiba, PR, Brazil
[b] Federal University of Paraná, Postgraduate Program in Sustainable Territorial Development, Coastal sector, Matinhos, PR, Brazil
[c] Federal University of Paraná, Department of Chemical Engineering, Curitiba, PR, Brazil
[d] Regional University of Blumenau, Department of Pharmaceutical Sciences, Blumenau, SC, Brazil
[E] Ceará State University, Laboratory for Bioprospecting and Monitoring of Natural Resources - LBMRN, Fortaleza, CE, Brazil

* *Corresponding author. E-mail: jenifer.araujo@ufpr.br*

Abstract: Essential oils from *Endlicheria paniculata* tissues were characterized by GC–MS, revealing a high concentration of sesquiterpenes. Major constituents were amorpha-4,9-dien-2-ol (41.12%) in leaves, β-muurola-4,10(14)-dien-1-ol (43.54%) in wood, and spathulenol (18.56%) in bark. In preliminary single-concentration screening assays, the leaf oil demonstrated preliminary in vitro inhibitory potential against α-glucosidase (98.44%) and acetylcholinesterase (AChE) (97.71%), alongside moderate larvicidal activity against *Aedes aegypti* ($LC_{50} = 61.56\ \mu g \cdot mL^{-1}$). Molecular docking simulations complemented these preliminary biological findings, showing that major leaf constituents, particularly amorpha-4,9-dien-2-ol and selina-3,7(11)-diene, exhibited favorable predicted binding interactions toward *Ae. aegypti* AChE-1, driven primarily by hydrophobic interactions. Overall, these findings provide theoretical insights and support further investigation of *E. paniculata* volatiles as potential candidates for vector control research and detailed bio-guided enzyme inhibition studies.

Keywords: Lauraceae; essential oil; sesquiterpenoids; enzyme inhibition; *Aedes aegypti;* molecular docking.

LIST OF CONTENTS

Figure S1. Annotated GC–MS total ion chromatogram (TIC) of the essential oil extracted from the wood of *Endlicheria paniculata*. Peak numbers correspond to compound identification numbers in Table 1.

Figure S2. Annotated GC–MS total ion chromatogram (TIC) of the essential oil extracted from the bark of *Endlicheria paniculata*. Peak numbers correspond to compound identification numbers in Table 2.

Figure S3. Annotated GC–MS total ion chromatogram (TIC) of the essential oil extracted from the dried leaves of *Endlicheria paniculata*. Peak numbers correspond to compound identification numbers in Table 3.

Figure S4. GC–MS total ion chromatogram (TIC) of the homologous n-alkane series (C_7--C_{30}) used for the calculation of linear retention indices (LRI) under identical chromatographic conditions.

Figure S5. 70 eV EI mass spectrum of compound 1 from the wood essential oil of *Endlicheria paniculata* (Figure 1A).

Figure S6. 70 eV EI mass spectrum of compound 2 from the wood essential oil of *Endlicheria paniculata* (Figure 1A).

Figure S7. 70 eV EI mass spectrum of compound 3 from the wood essential oil of *Endlicheria paniculata* (Figure 1A).

Figure S8. 70 eV EI mass spectrum of compound 4 from the wood essential oil of *Endlicheria paniculata* (Figure 1A).

Figure S9. 70 eV EI mass spectrum of compound 5 from the wood essential oil of *Endlicheria paniculata* (Figure 1A).

Figure S10. 70 eV EI mass spectrum of compound 6 from the wood essential oil of *Endlicheria paniculata* (Figure 1A).

Figure S11. 70 eV EI mass spectrum of compound 7 from the bark essential oil of *Endlicheria paniculata* (Figure 1B).

Figure S12. 70 eV EI mass spectrum of compound 8 from the bark essential oil of *Endlicheria paniculata* (Figure 1B).

Figure S13. 70 eV EI mass spectrum of compound 9 from the bark essential oil of *Endlicheria paniculata* (Figure 1B).

Figure S14. 70 eV EI mass spectrum of compound 10 from the bark essential oil of *Endlicheria paniculata* (Figure 1B).

Figure S15. 70 eV EI mass spectrum of compound 11 from the bark essential oil of *Endlicheria paniculata* (Figure 1B).

Figure S16. 70 eV EI mass spectrum of compound 12 from the bark essential oil of *Endlicheria paniculata* (Figure 1B).

Figure S17. 70 eV EI mass spectrum of compound 13 from the leaf essential oil of *Endlicheria paniculata* (Figure 1C).

Figure S18. 70 eV EI mass spectrum of compound 14 from the leaf essential oil of *Endlicheria paniculata* (Figure 1C).

Figure S19. 70 eV EI mass spectrum of compound 15 from the leaf essential oil of *Endlicheria paniculata* (Figure 1C).

Figure S20. 70 eV EI mass spectrum of compound 16 from the leaf essential oil of *Endlicheria paniculata* (Figure 1C).

Figure S21. 70 eV EI mass spectrum of compound 17 from the leaf essential oil of *Endlicheria paniculata* (Figure 1C).

Figure S22. 70 eV EI mass spectrum of compound 18 from the leaf essential oil of *Endlicheria paniculata* (Figure 1C).

Table S1. Detailed chromatographic and mass spectrometric identification parameters for essential oil constituents of *Endlicheria paniculata* tissues**.**

Table S2. Chemical traceability, PubChem database identifiers, SMILES strings, InChIKeys, and protonation states of all major essential oil constituents of *Endlicheria paniculata* and reference controls evaluated in molecular docking simulations.

Table S3. Target proteins, PDB access codes, grid box coordinates (x, y, z), dimensions (AA), and binding free energies (ΔG, kcal/mol) calculated for major constituents of *Endlicheria paniculata* and reference ligands.

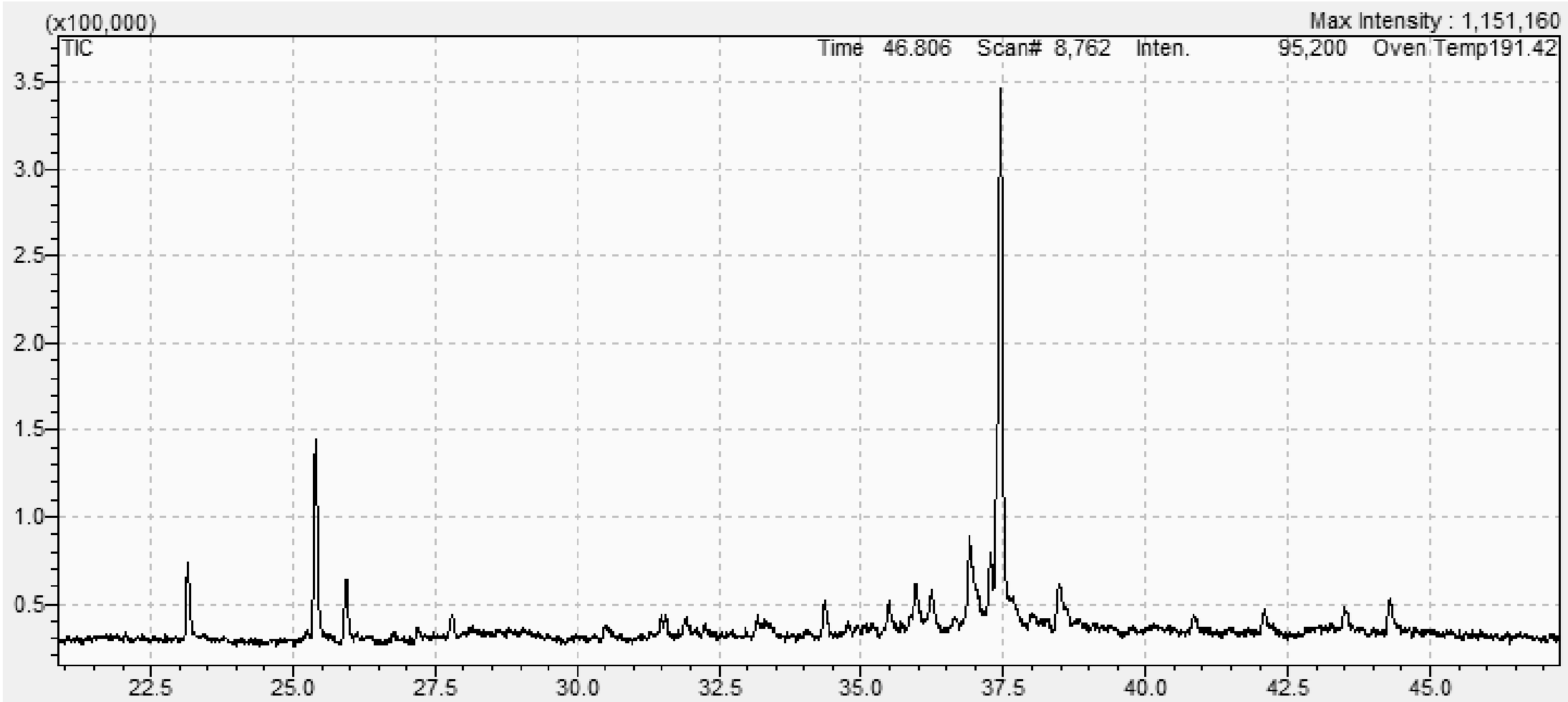

Figure S1. Annotated GC–MS total ion chromatogram (TIC) of the essential oil extracted from the wood of *E. paniculata*. Peak numbers correspond to compound identification numbers in Table 1.

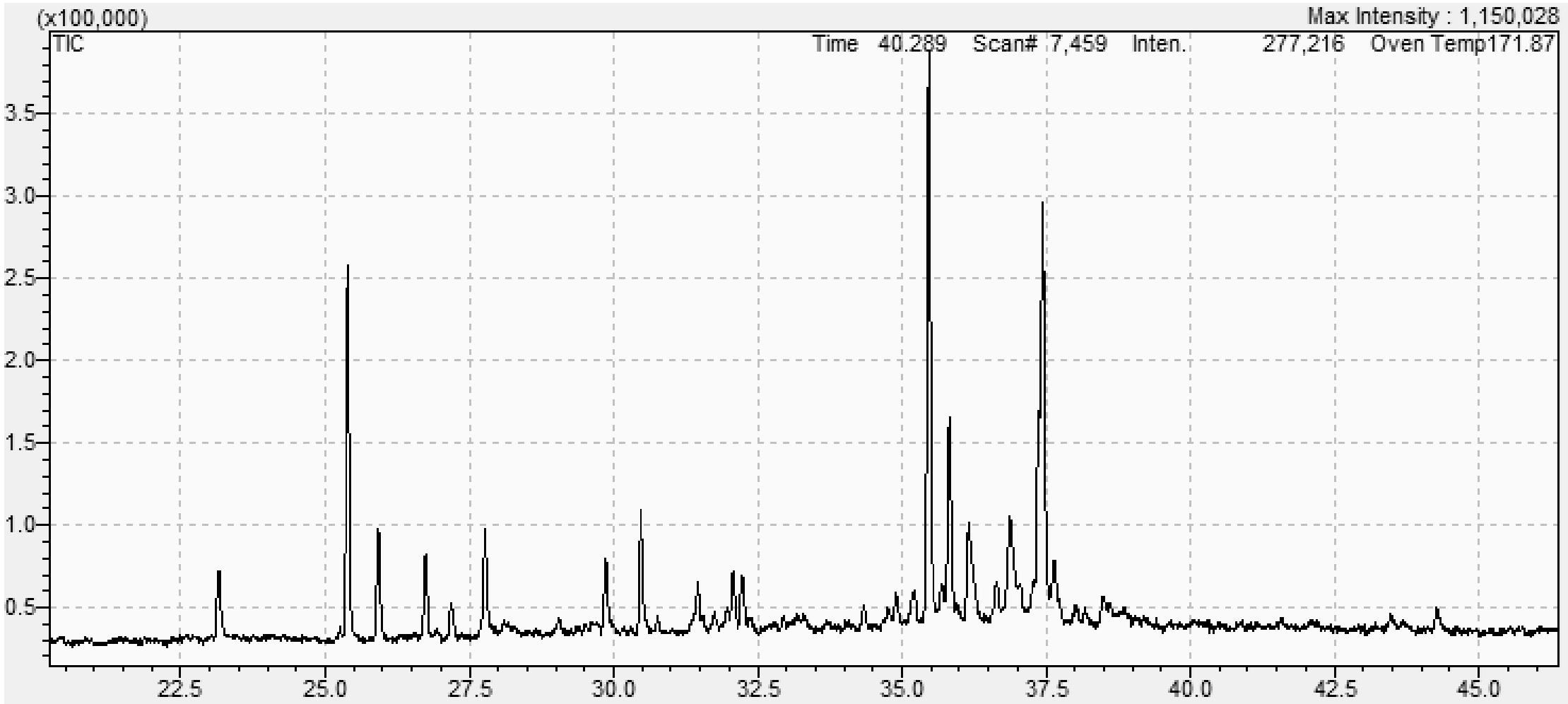

Figure S2. Annotated GC–MS total ion chromatogram (TIC) of the essential oil extracted from the bark of *E. paniculata*. Peak numbers correspond to compound identification numbers in Table 2.

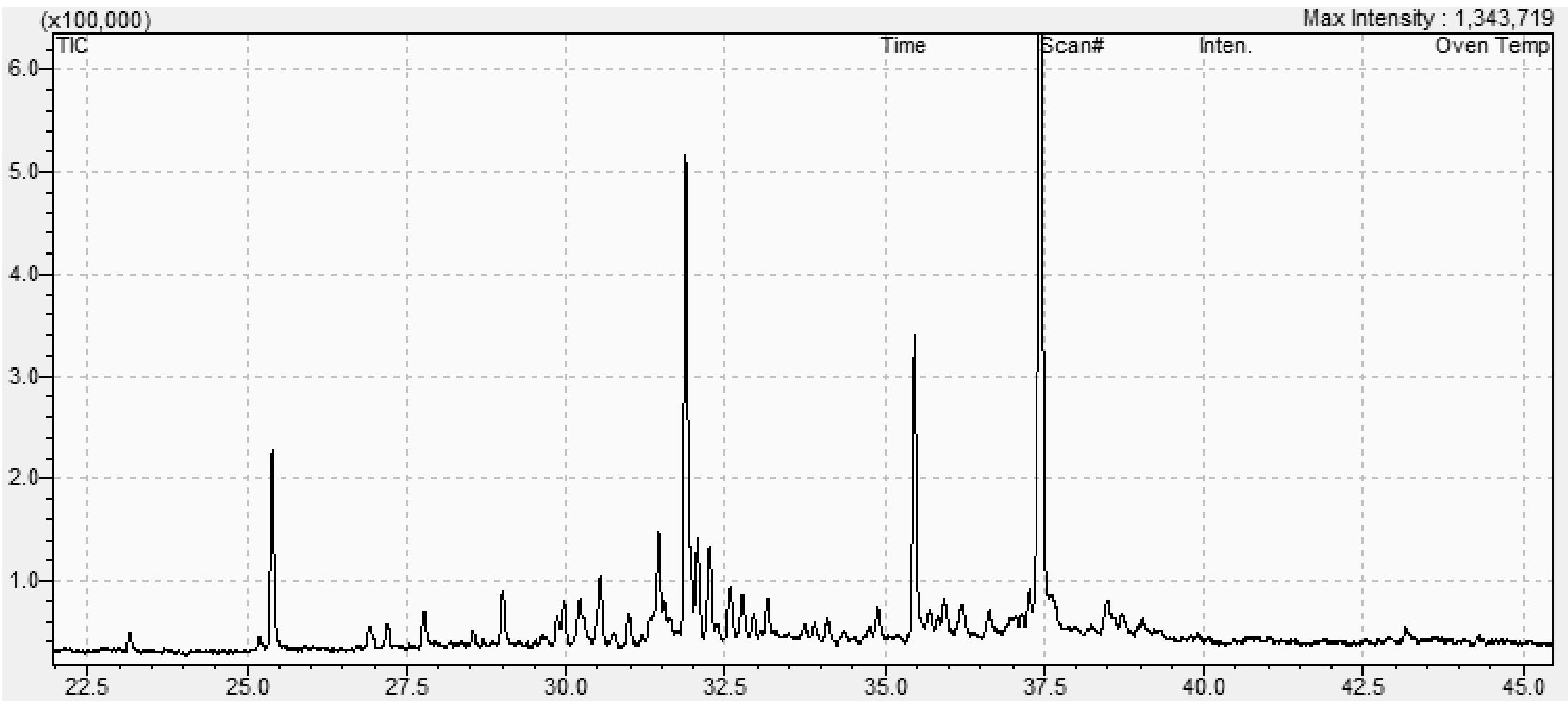

Figure S3. Annotated GC–MS total ion chromatogram (TIC) of the essential oil extracted from the dried leaves of *E. paniculata*. Peak numbers correspond to compound identification numbers in Table 3.

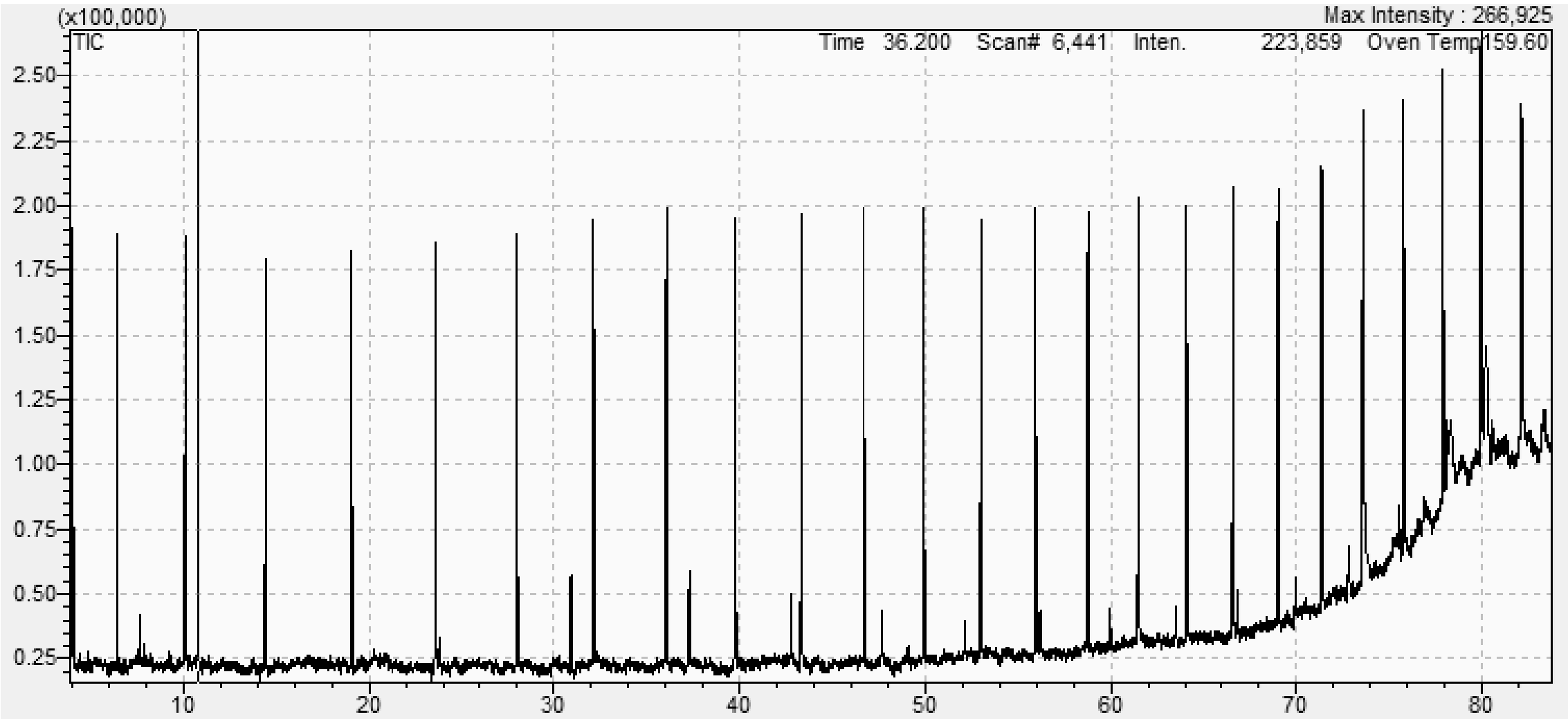

Figure S4. GC–MS total ion chromatogram (TIC) of the homologous n-alkane series (C_7--C_{30}) used for the calculation of linear retention indices (LRI) under identical chromatographic conditions applied to the essential oil analyses.

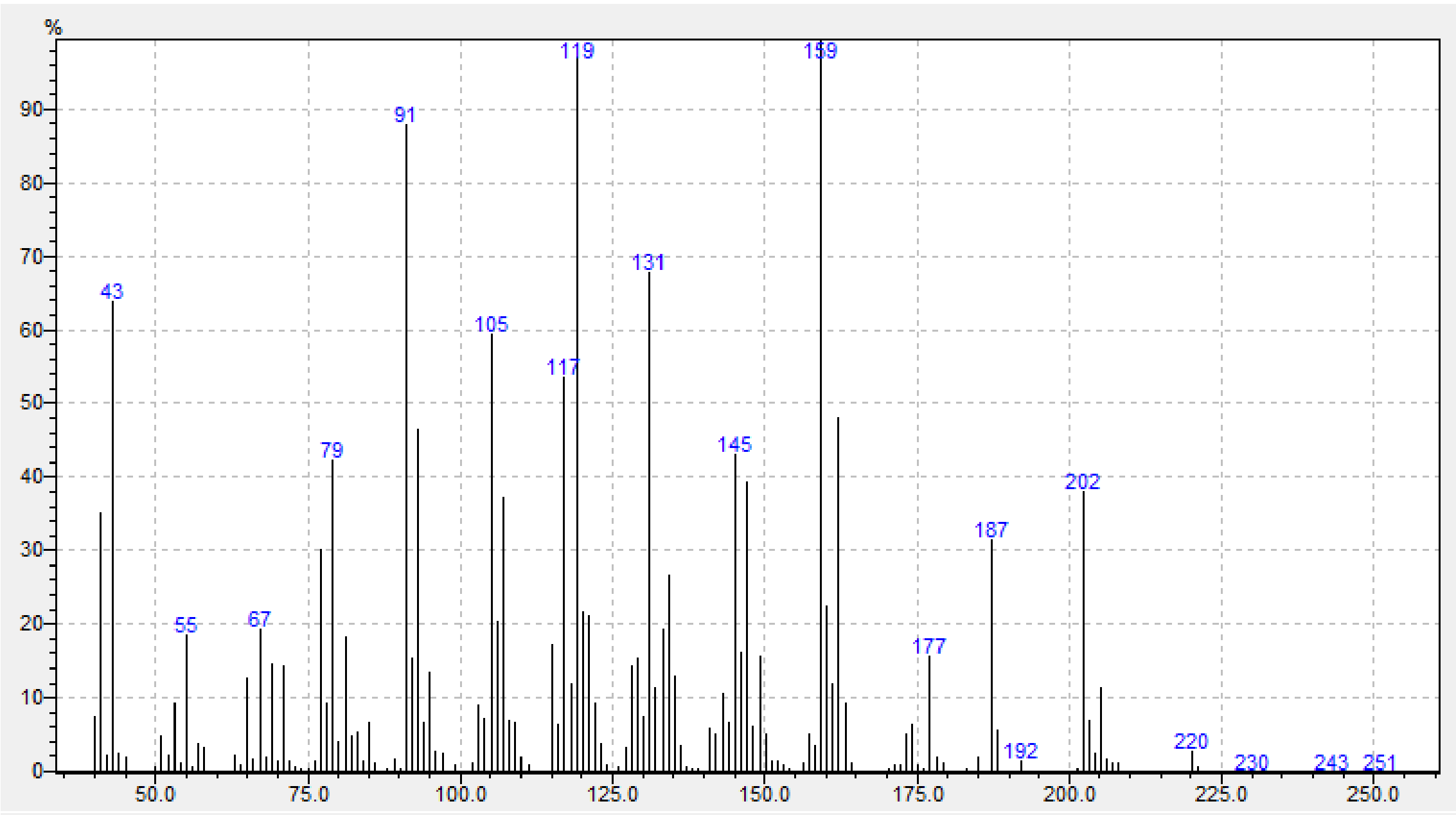

Figure S5. 70 eV EI mass spectrum of compound **1** from the wood essential oil of *Endlicheria paniculata* (Figure 1A).

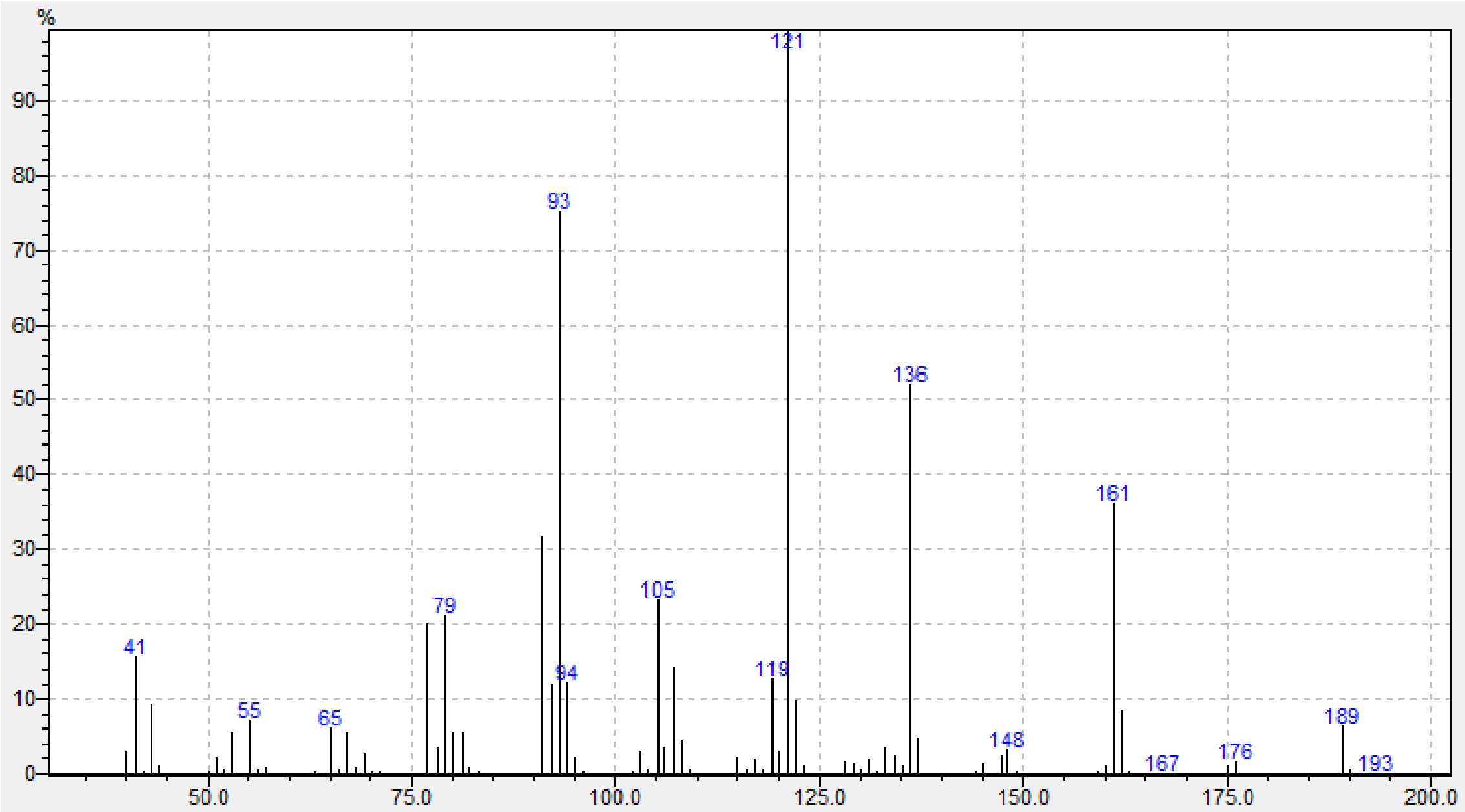

Figure S6. 70 eV EI mass spectrum of compound 2 from the wood essential oil of *Endlicheria paniculata* (Figure 1A).

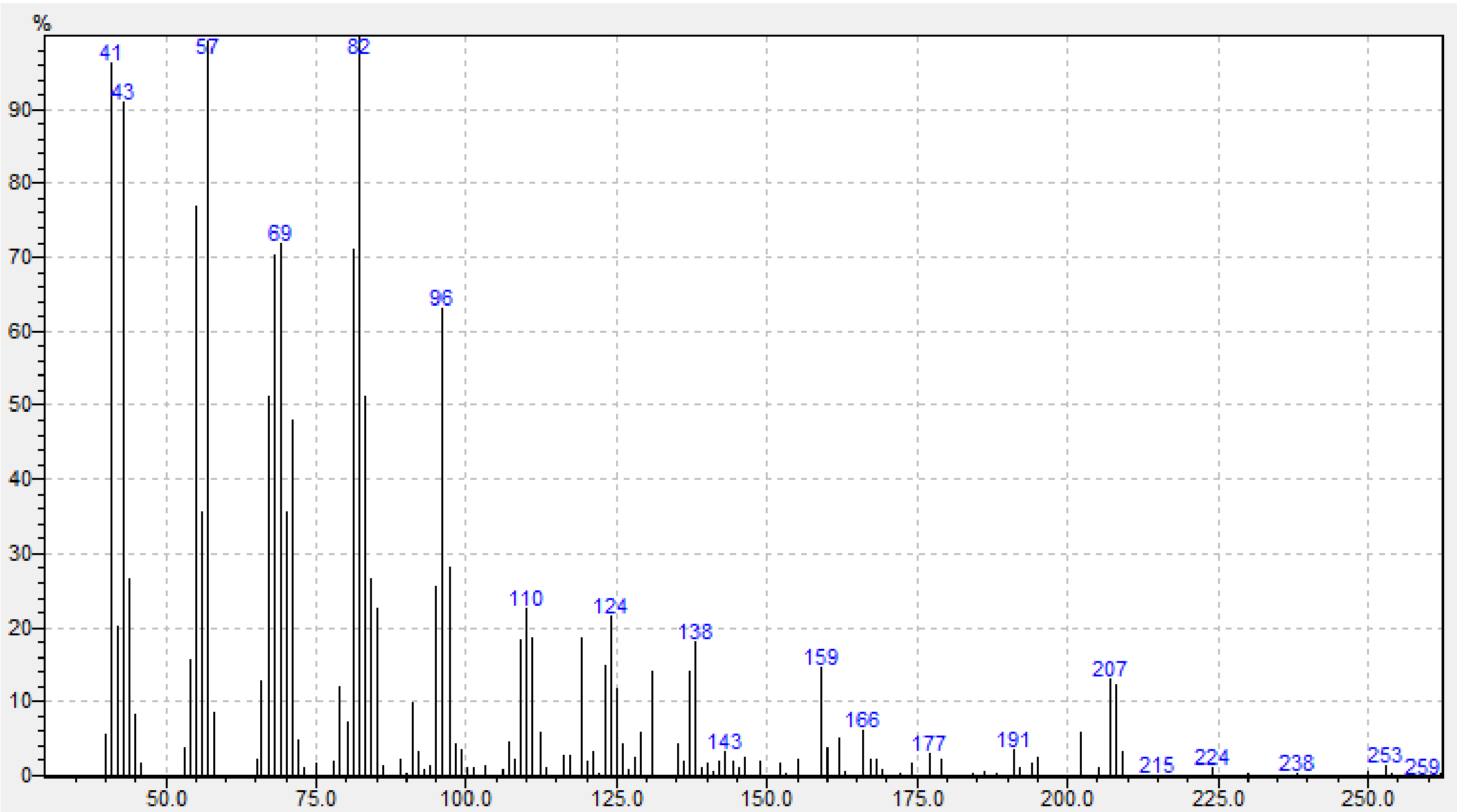

Figure S7. 70 eV EI mass spectrum of compound 3 from the wood essential oil of *Endlicheria paniculata* (Figure 1 A).

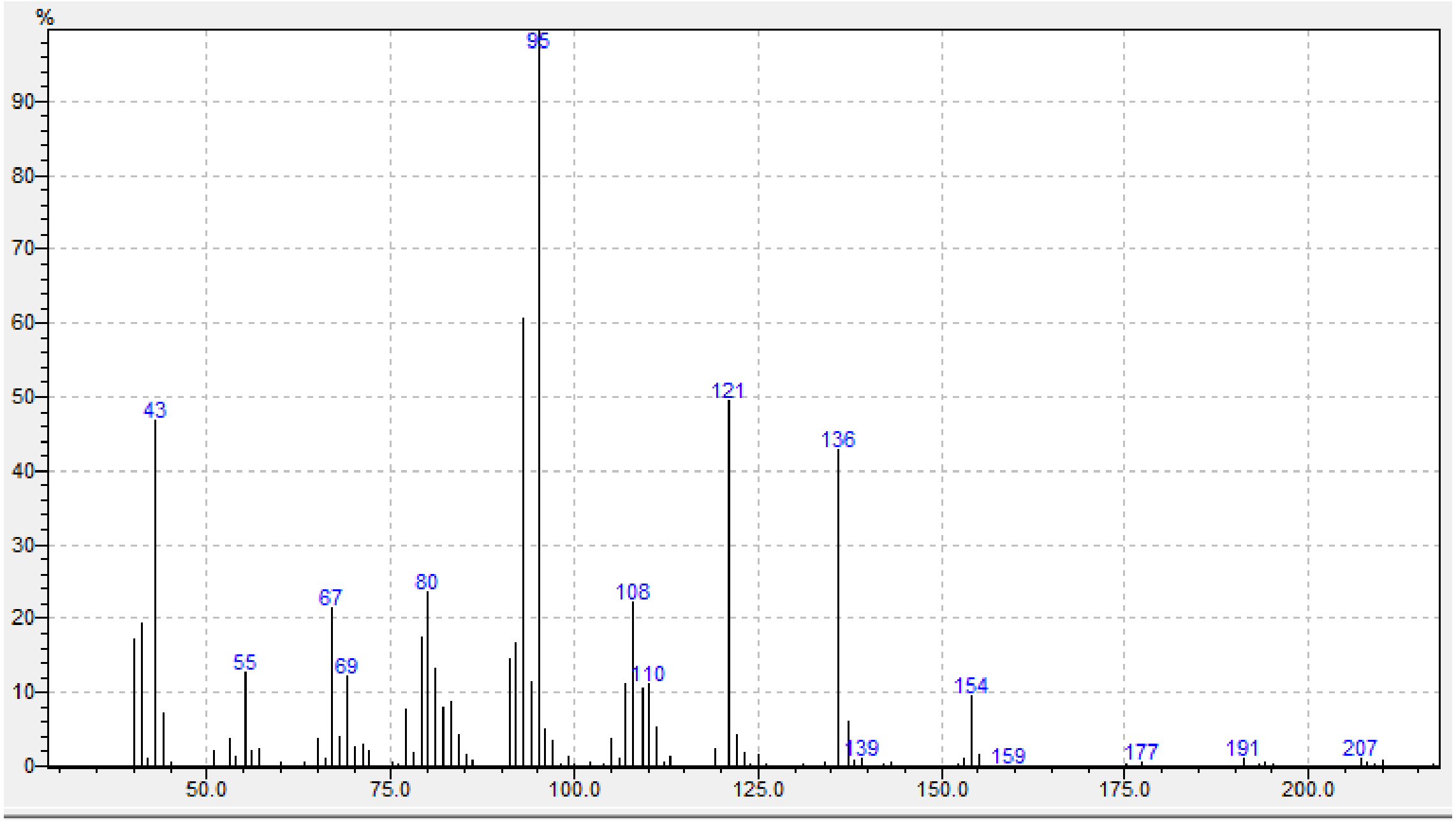

Figure S8. 70 eV EI mass spectrum of compound 4 from the wood essential oil of *Endlicheria paniculata* (Figure 1 A).

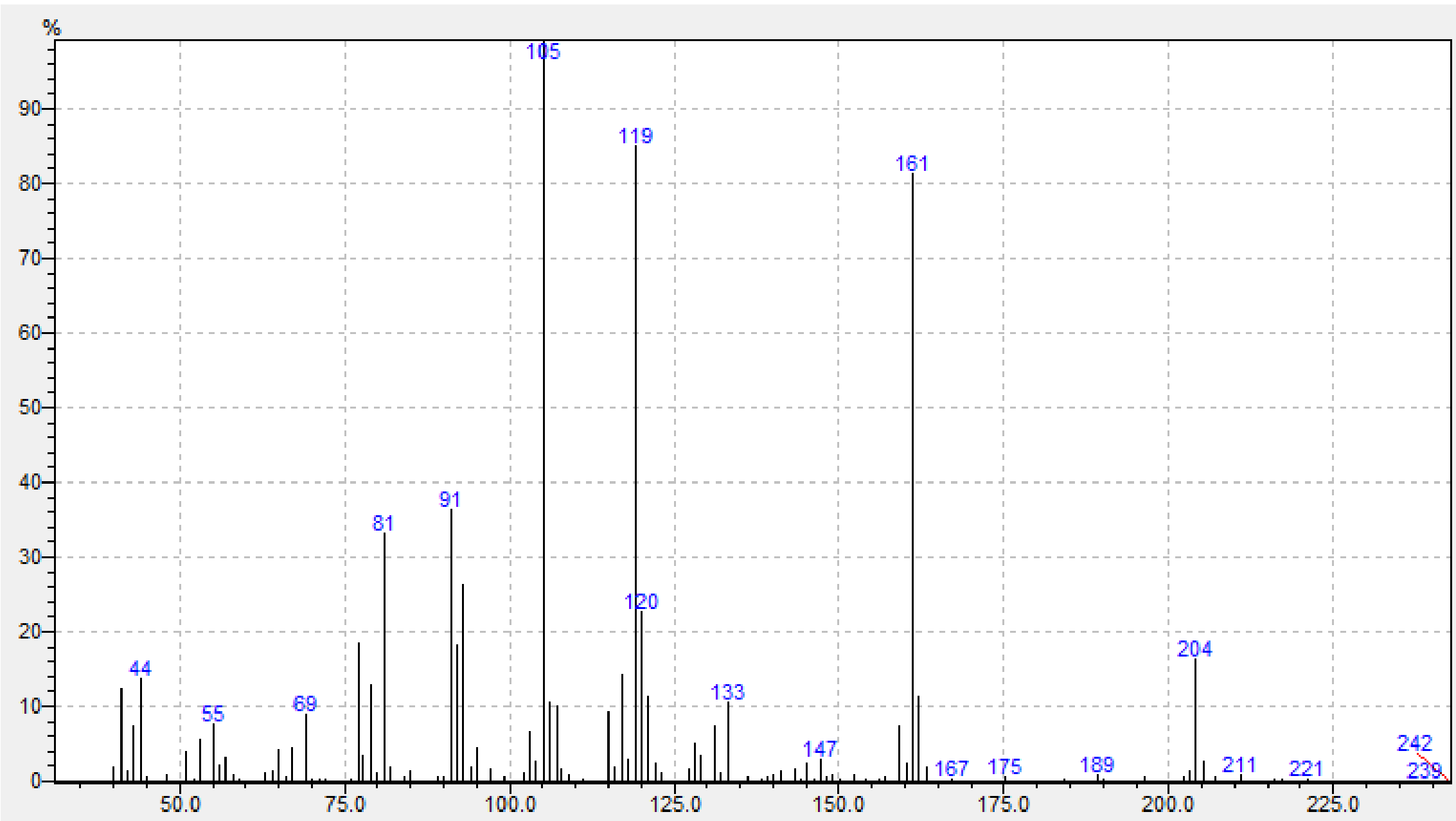

Figure S9. 70 eV EI mass spectrum of compound 5 from the wood essential oil of *Endlicheria paniculata* (Figure 1A).

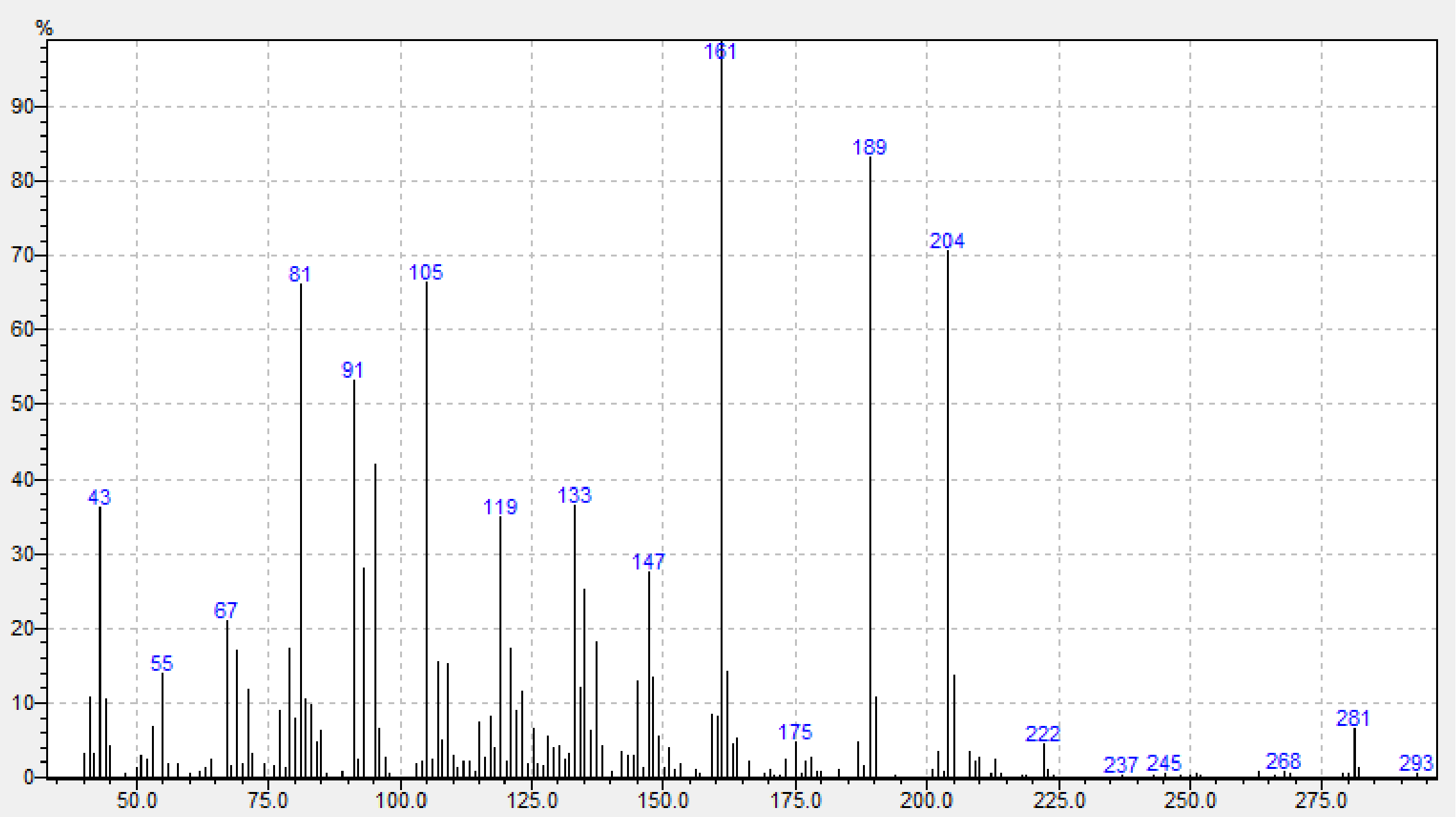

Figure S10. 70 eV EI mass spectrum of compound 6 from the wood essential oil of *Endlicheria paniculata* (Figure 1 A).

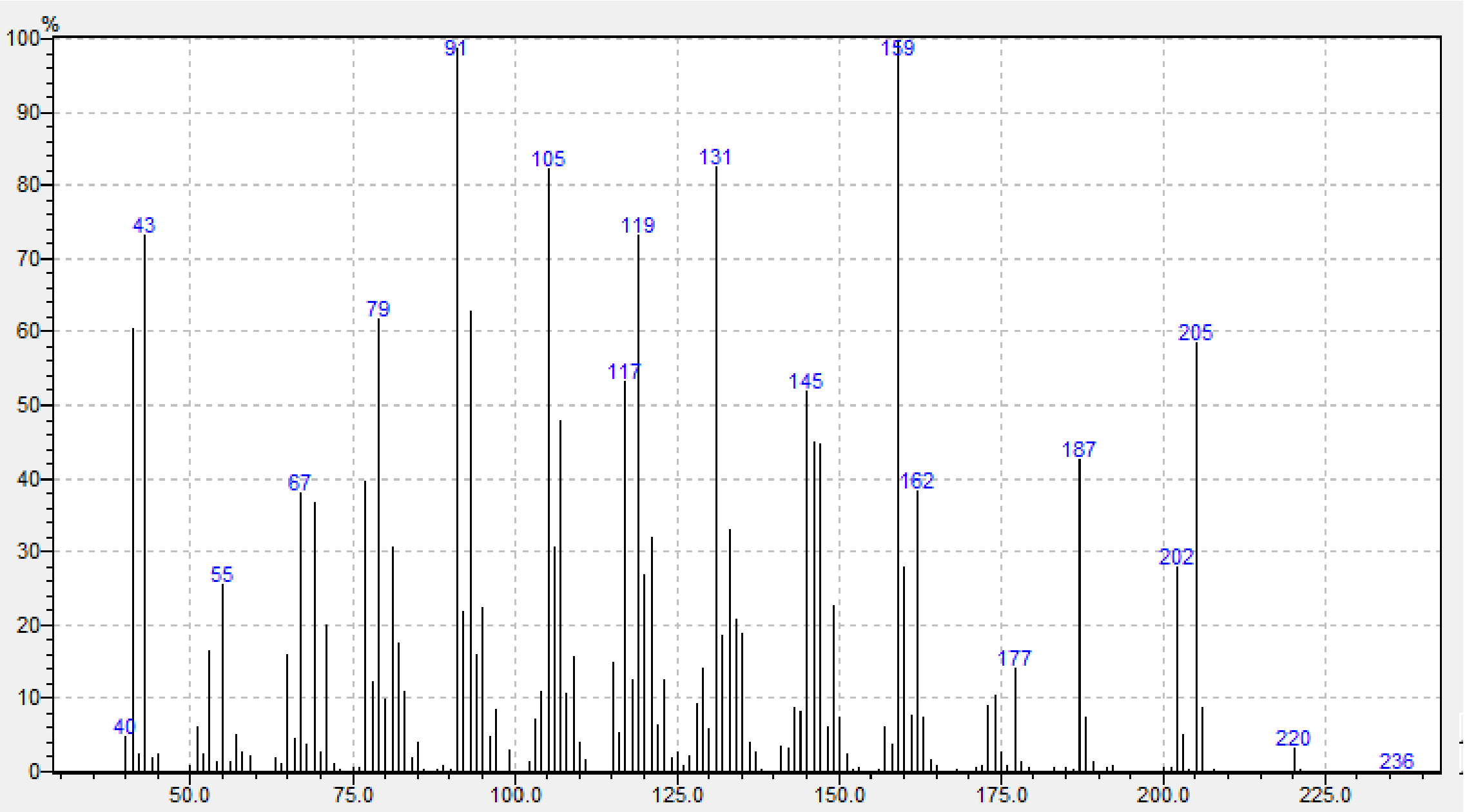

Figure S11. 70 eV EI mass spectrum of compound 7 from the bark essential oil of *Endlicheria paniculata* (Figure 1B).

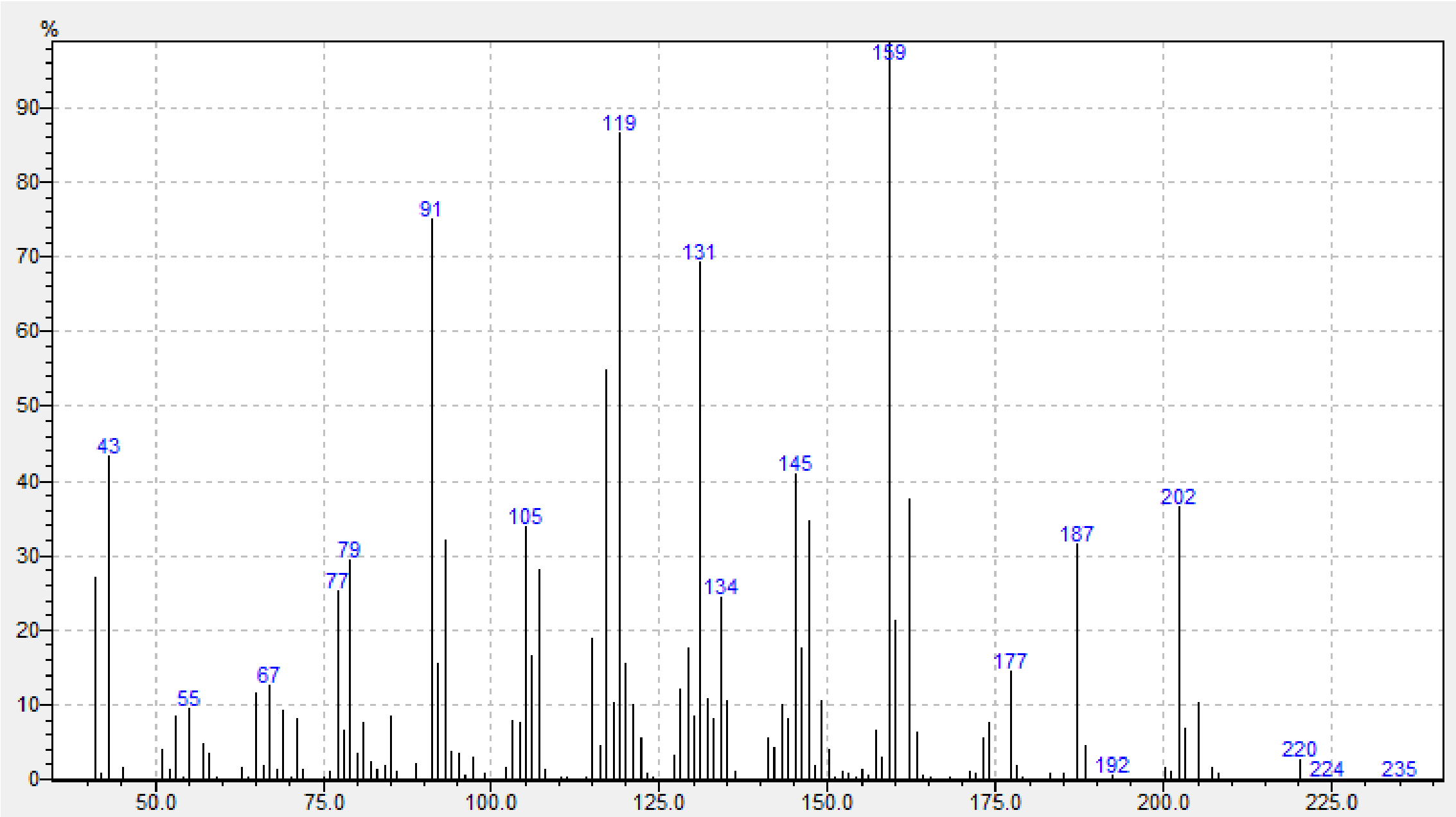

Figure S12. 70 eV EI mass spectrum of compound 8 from the bark essential oil of *Endlicheria paniculata* (Figure 1B).

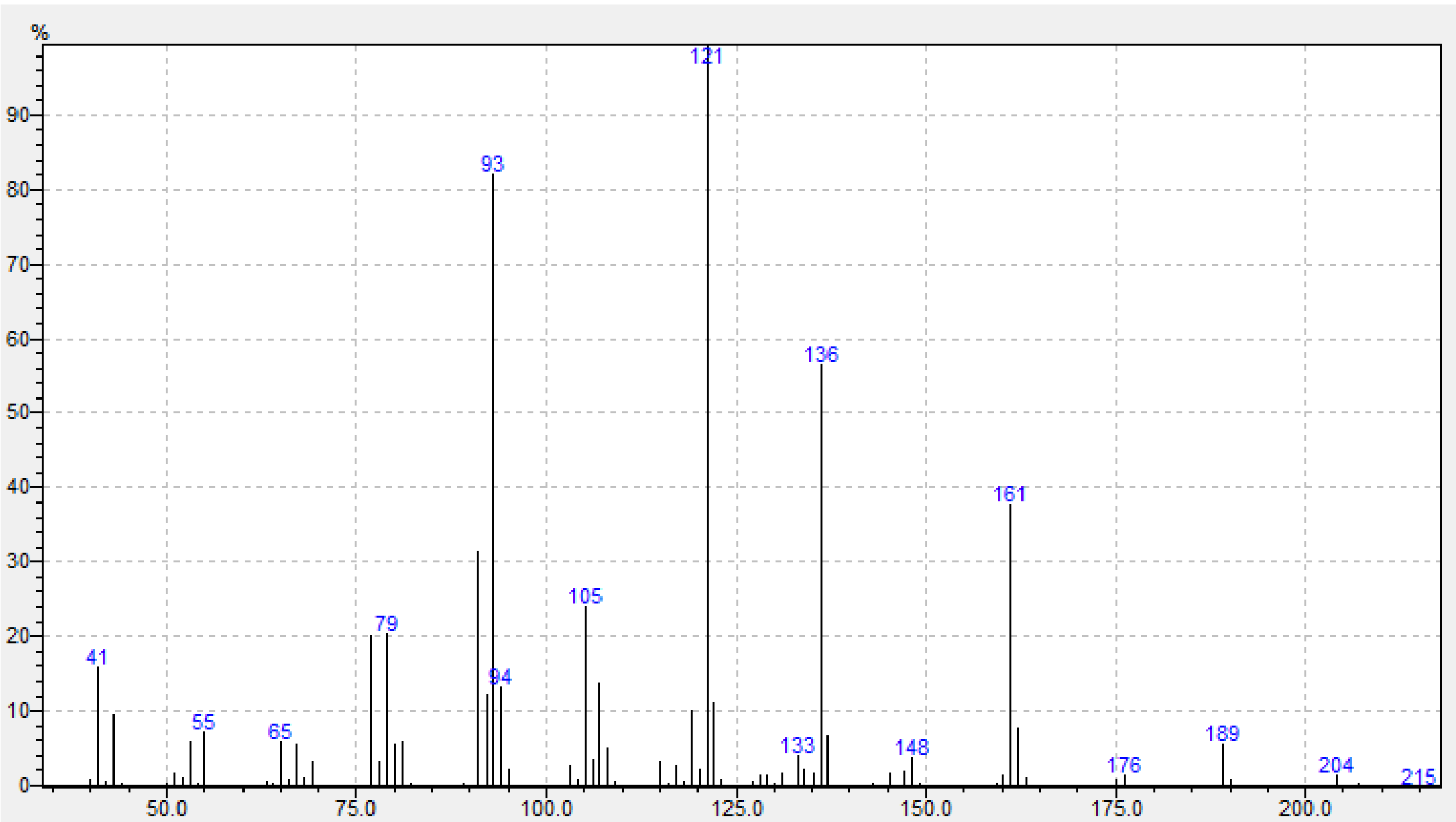

Figure S13. 70 eV EI mass spectrum of compound 9 from the bark essential oil of *Endlicheria paniculata* (Figure 1 B).

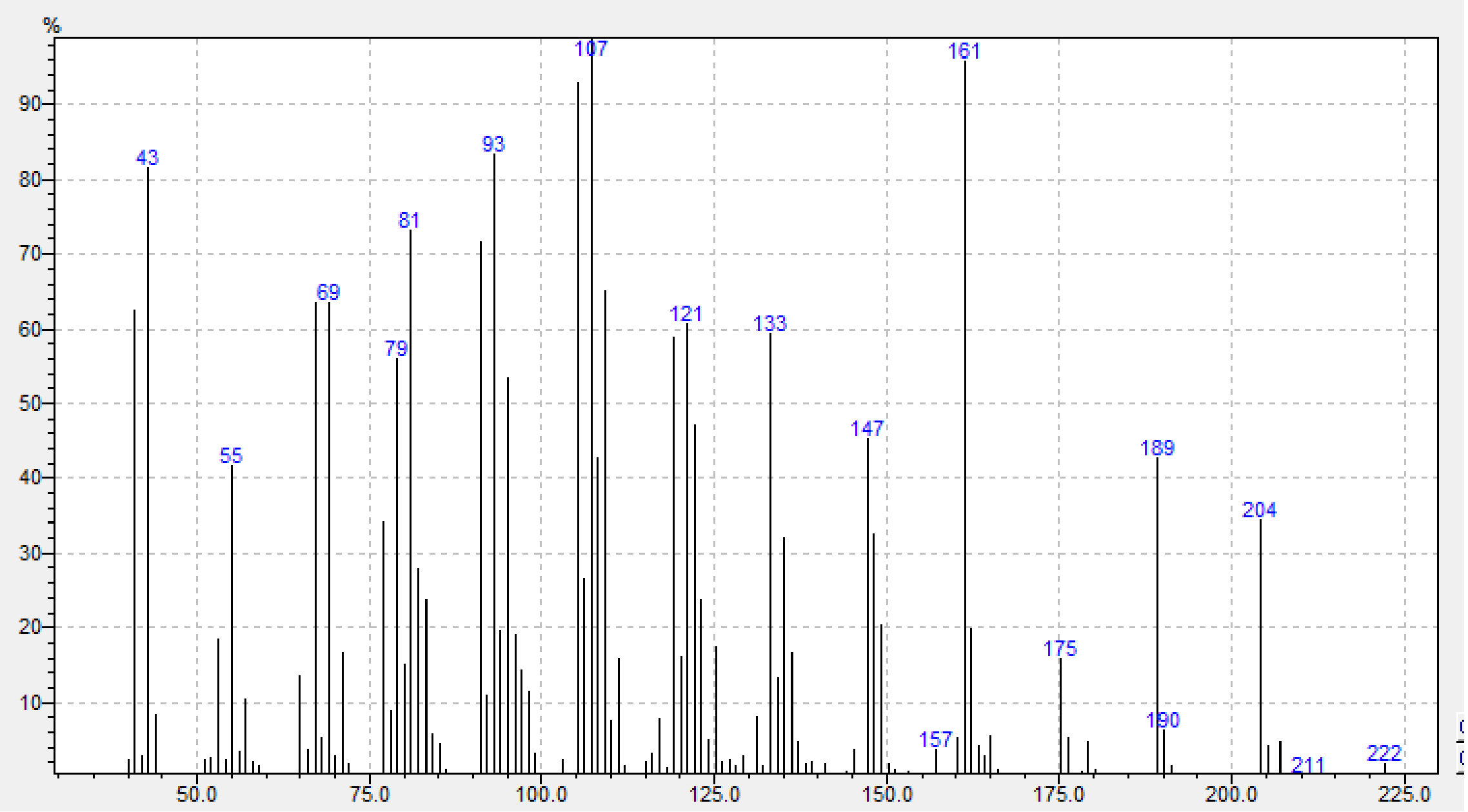

Figure S14. 70 eV EI mass spectrum of compound 10 from the bark essential oil of *Endlicheria paniculata* (Figure 1B).

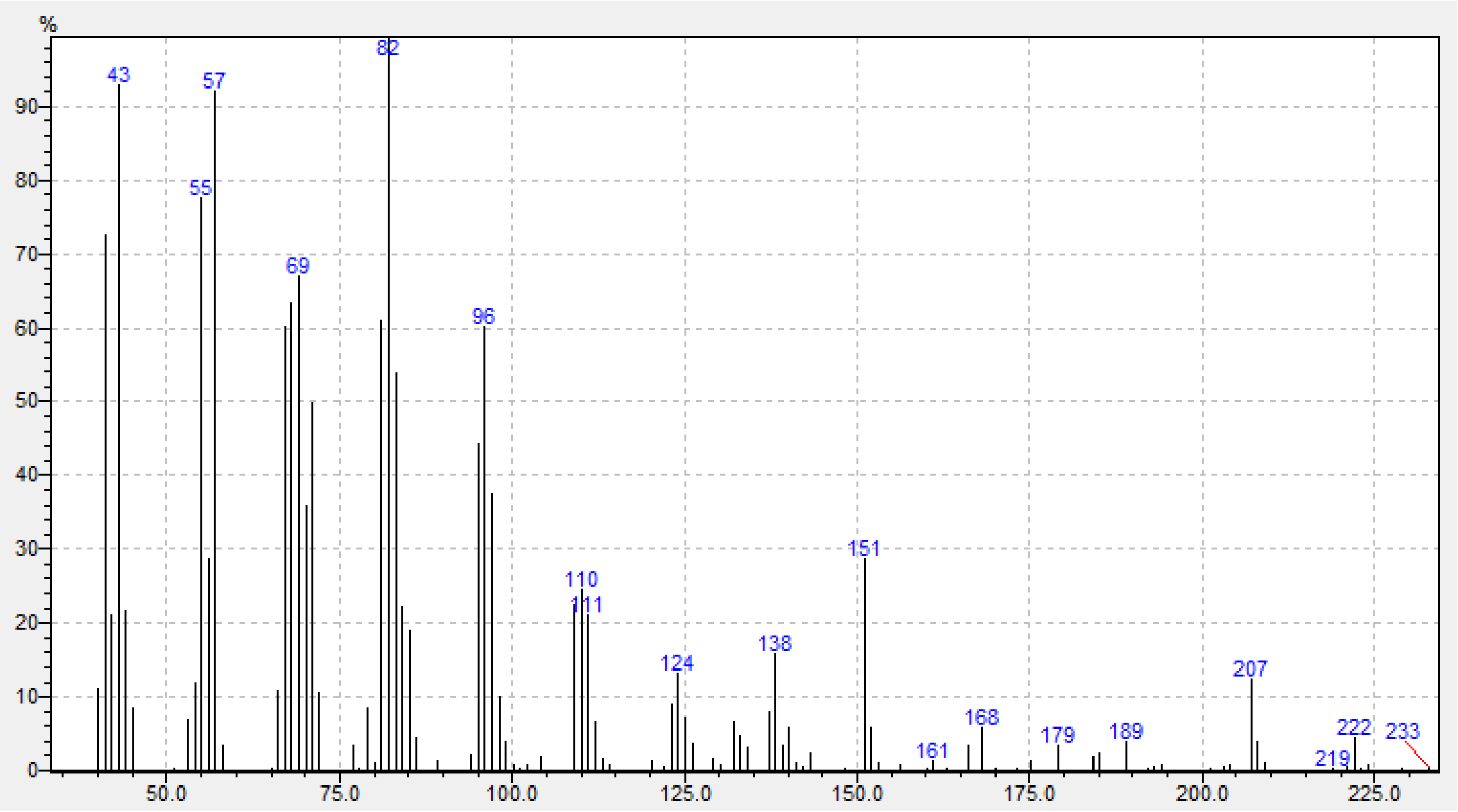

Figure S15. 70 eV EI mass spectrum of compound 11 from the bark essential oil of *Endlicheria paniculata* (Figure 1B).

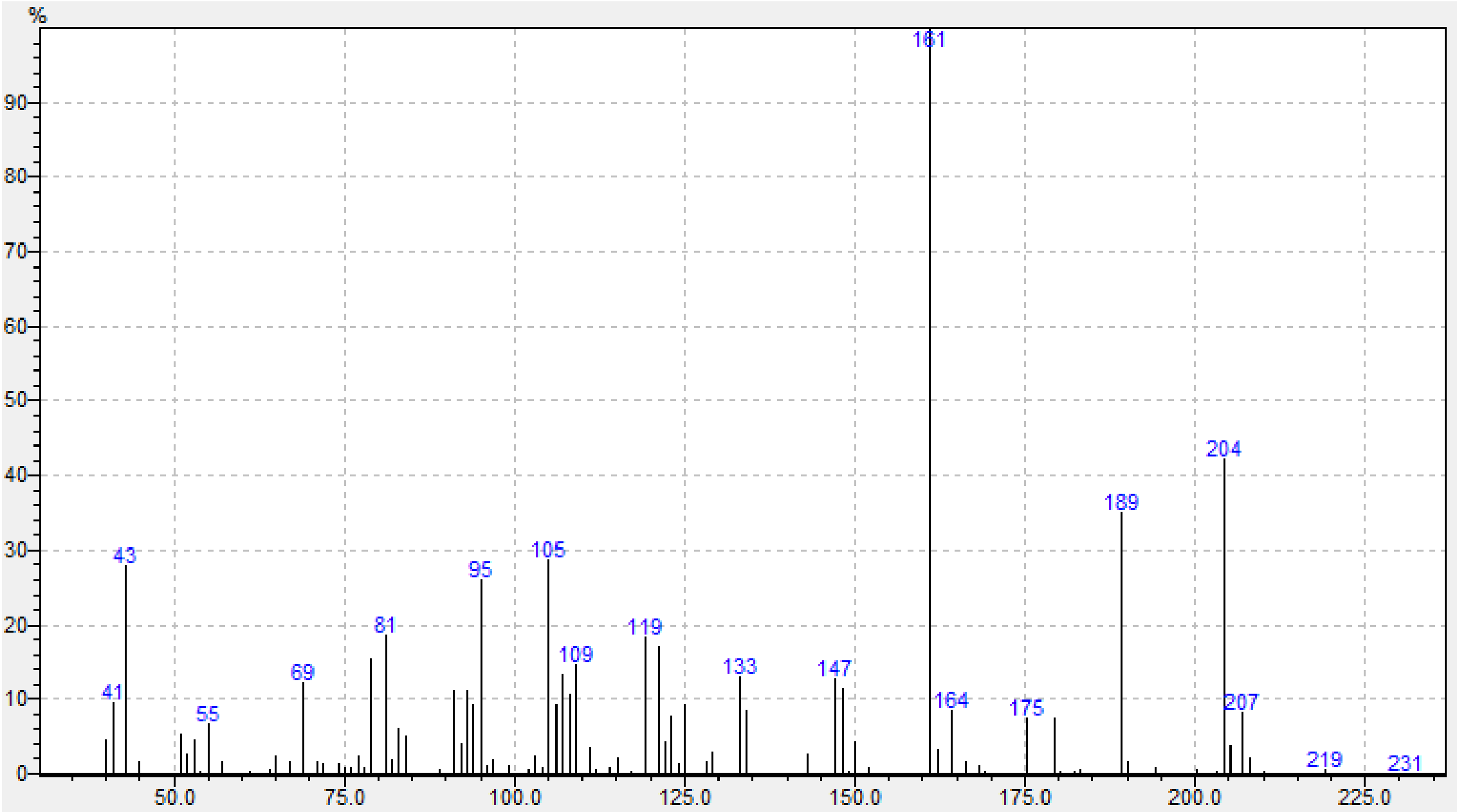

Figure S16. 70 eV EI mass spectrum of compound 12 from the bark essential oil of *Endlicheria paniculata* (Figure 1 B).

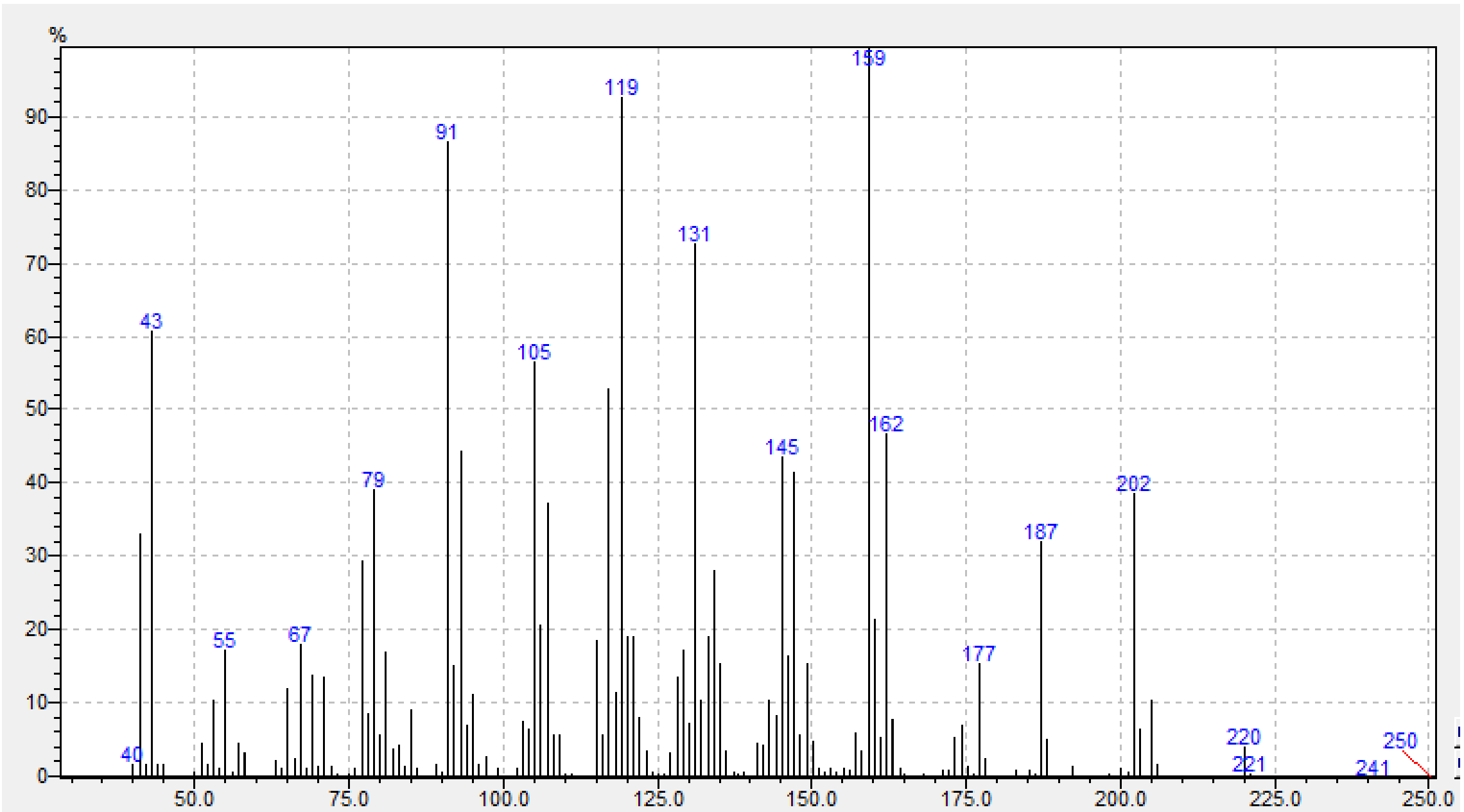

Figure S17. 70 eV EI mass spectrum of compound 13 from the leaves essential oil of *Endlicheria paniculata* (Figure 1 C).

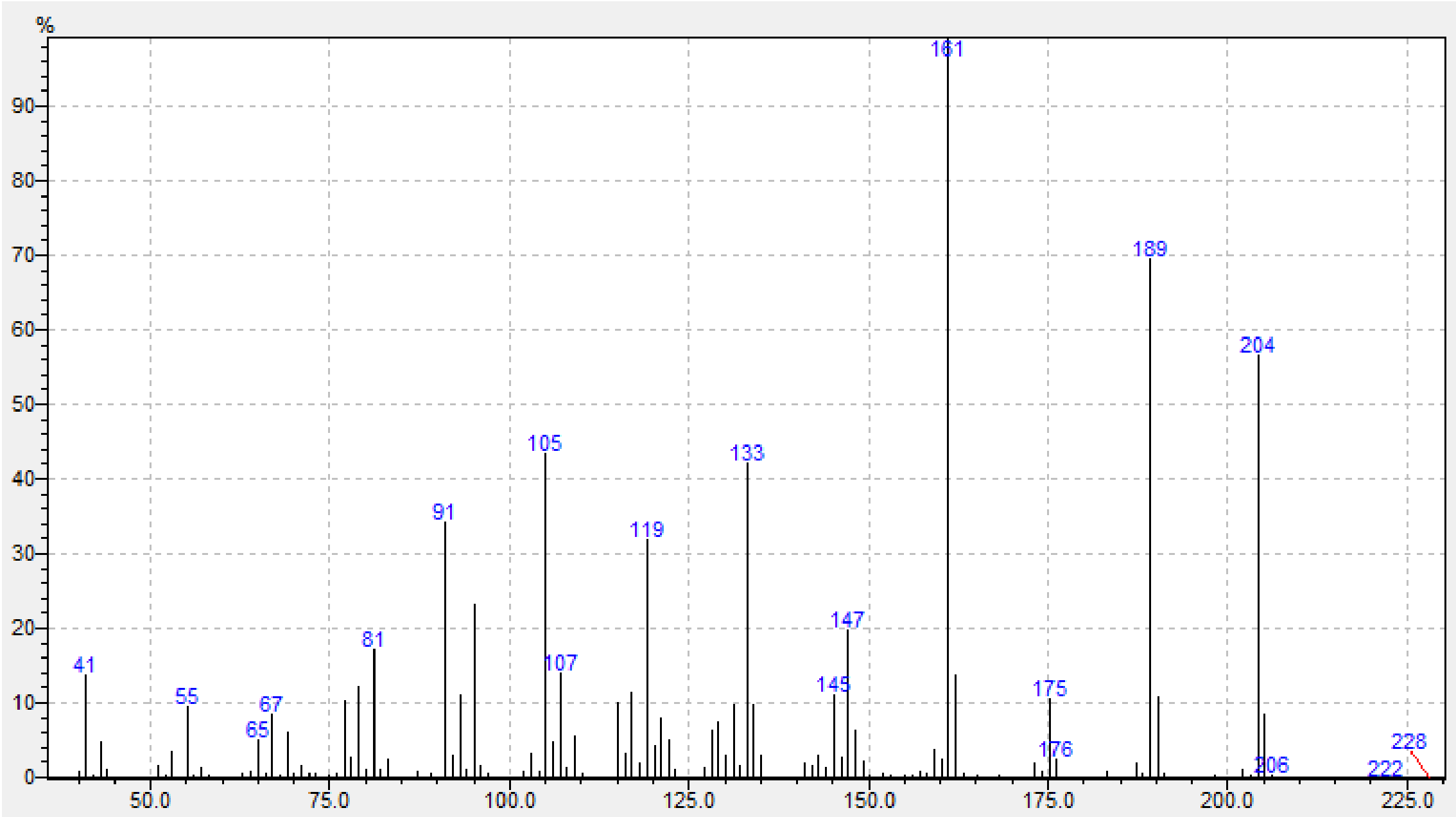

Figure S18. 70 eV EI mass spectrum of compound 14 from the leaves essential oil of *Endlicheria paniculata* (Figure 1 C).

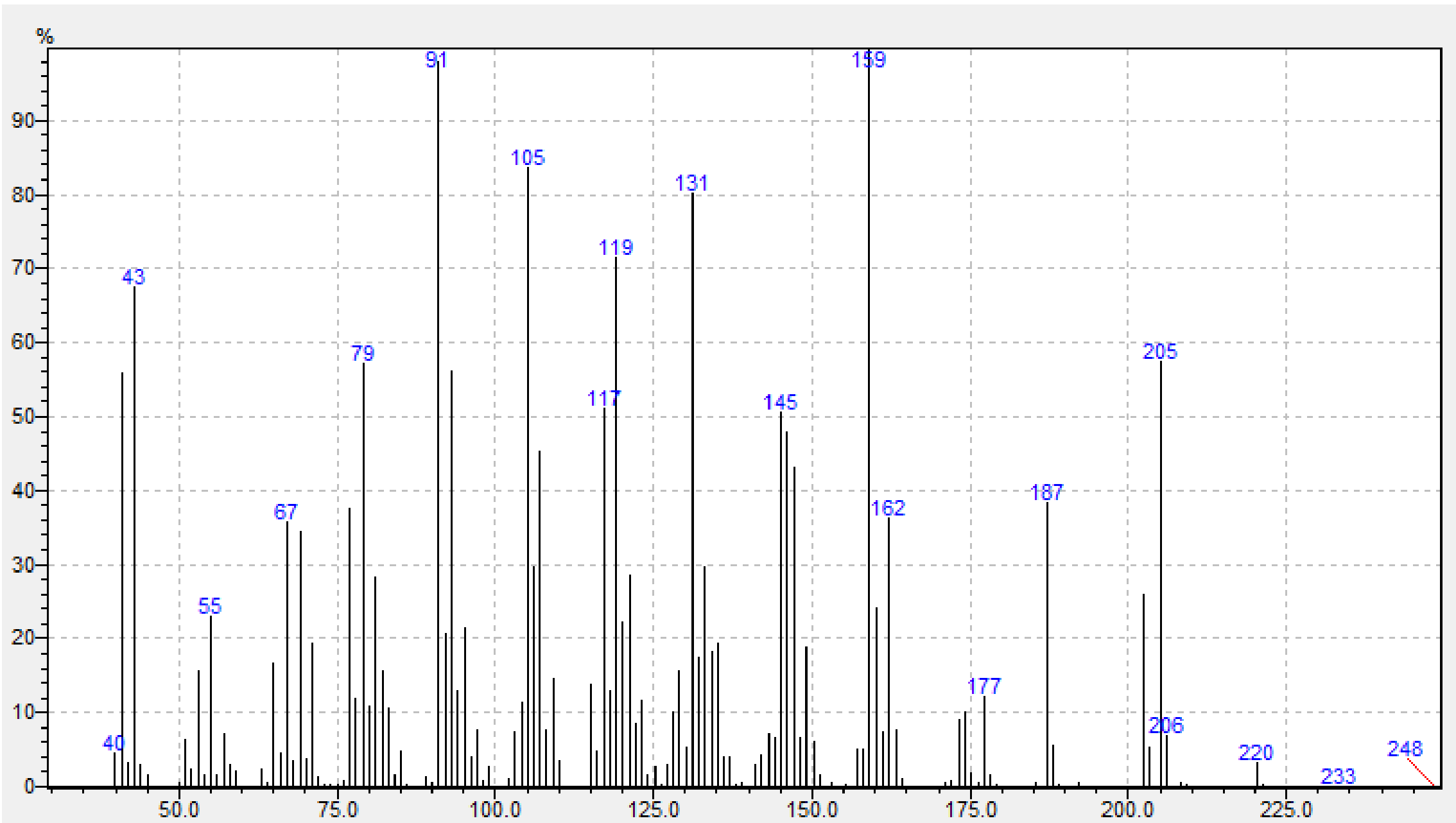

Figure S19. 70 eV EI mass spectrum of compound 15 from the leaves essential oil of *Endlicheria paniculata* (Figure 1C).

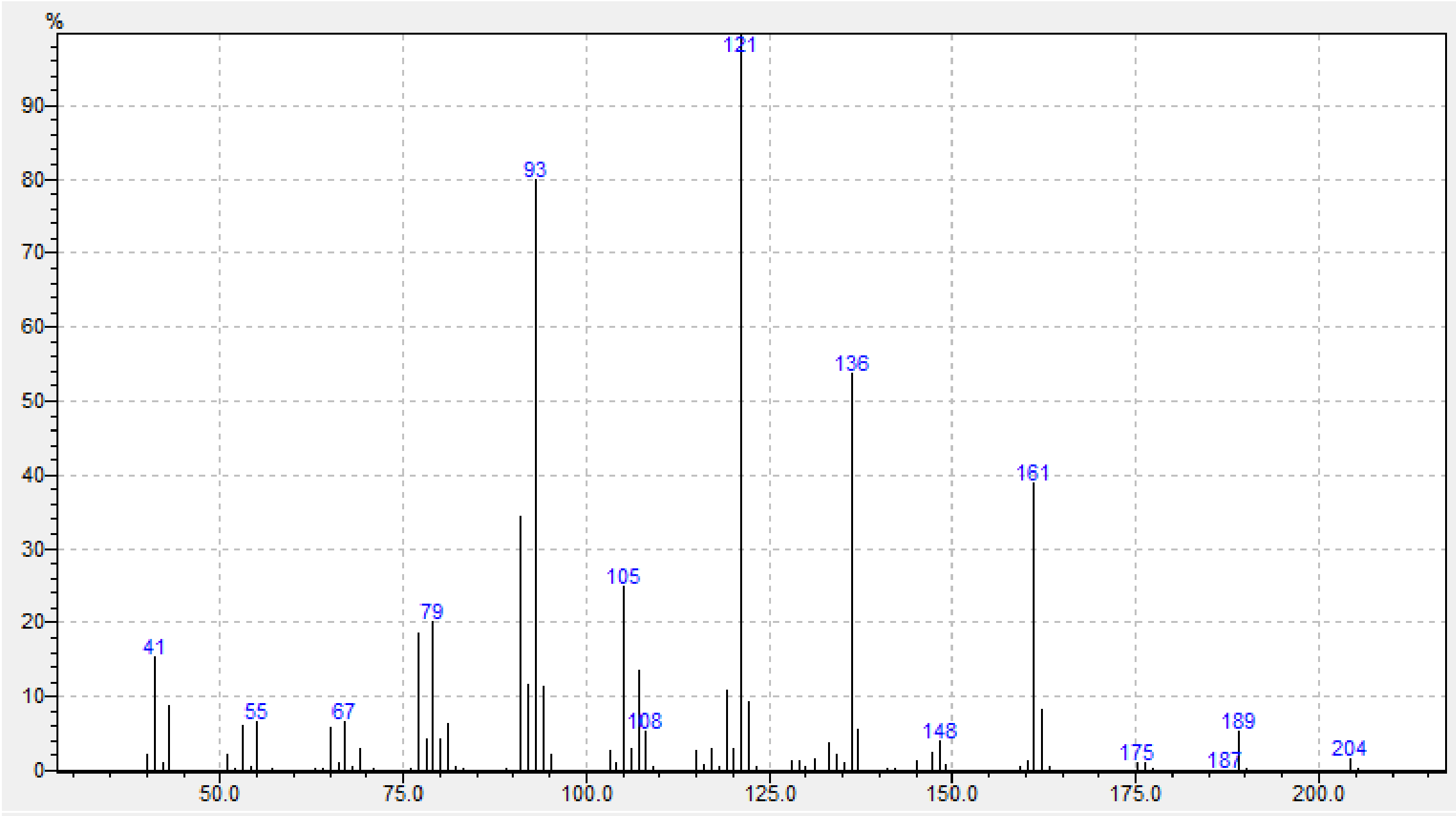

Figure S20. 70 eV EI mass spectrum of compound 16 from the leaves essential oil of *Endlicheria paniculata* (Figure 1 C).

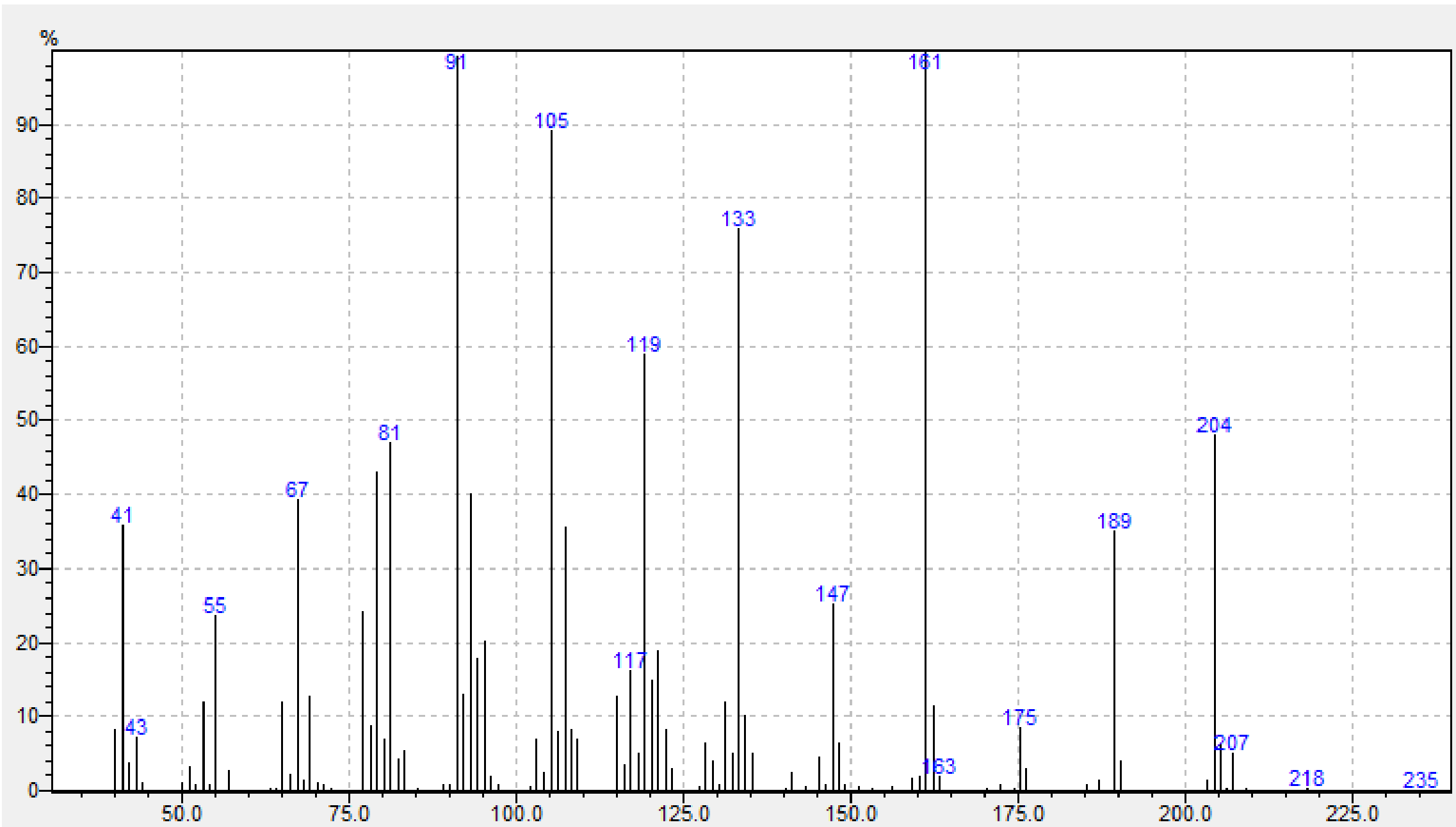

Figure S21. 70 eV EI mass spectrum of compound 17 from the leaves essential oil of *Endlicheria paniculata* (Figure 1C).

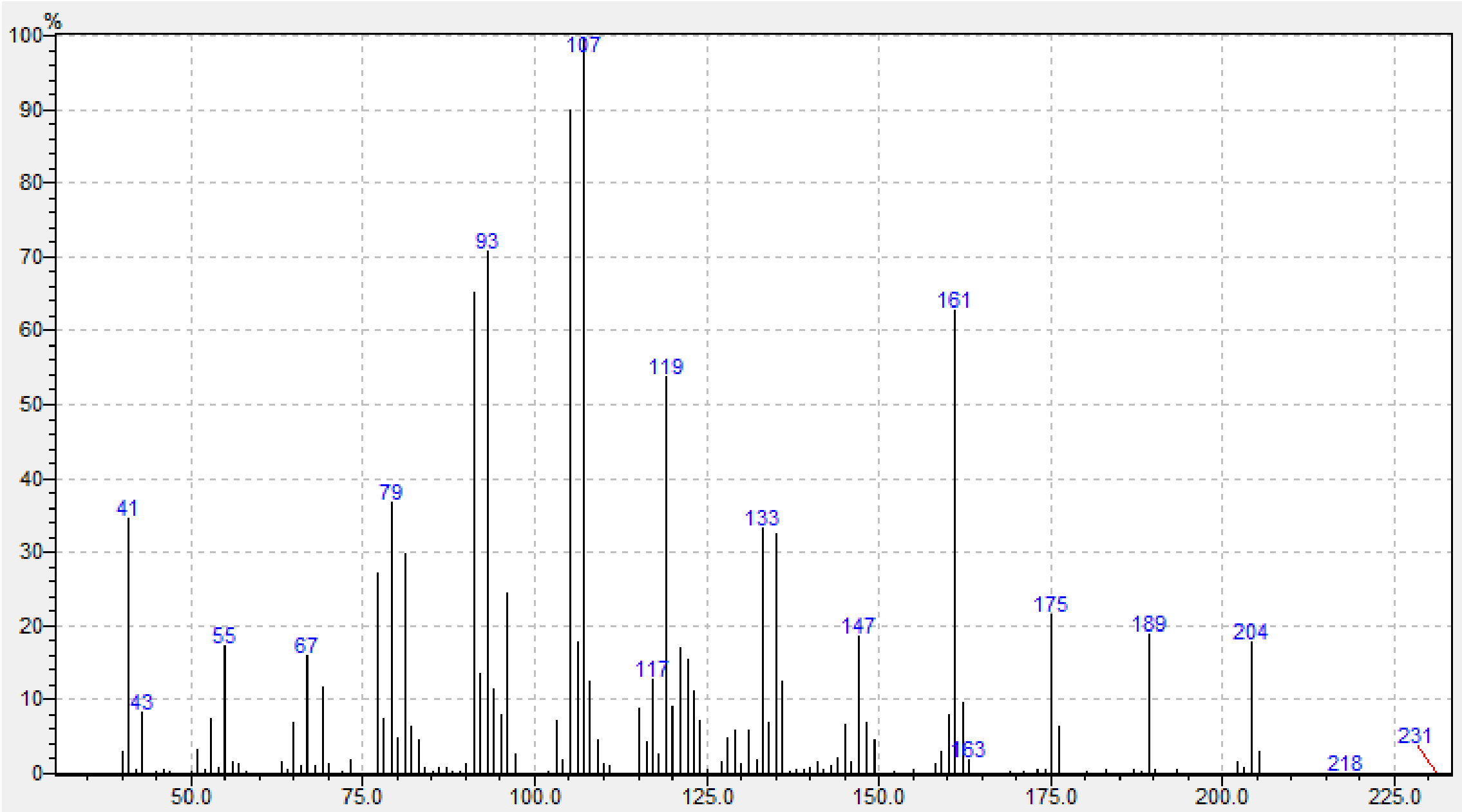

Figure S22. 70 eV EI mass spectrum of compound 18 from the leaves essential oil of *Endlicheria paniculata* (Figure 1C).

Table S1. Detailed chromatographic and mass spectrometric identification parameters for essential oil constituents of *Endlicheria paniculata* tissues.

Nº	Compound	Tissue	R_t (min)	RI_{calc}	RI_{lit}	Key Diagnostic Ions (m/z, base peak in bold)	Match Score (%)
1	Sabinene	Leaves	7.865	969	987	93, 91, 77, 69, 41	96
2	δ-Elemene	Wood/Bark	25.500	1405	1403	121, 161, 93, 105, 41	94
3	γ-Elemene	Leaves	25.480	1434	1403	121, 161, 107, 93, 41	95
4	Dehydro-aromadendrane	Leaves	27.845	1460	1461	161, 105, 119, 91, 202 (M^+)	93
5	γ-Gurjunene	Leaves	29.115	1475	1492	161, 105, 119, 91, 204 (M^+)	92
6	Viridiflorene	Leaves	29.905	1496	1512	161, 105, 120, 91, 204 (M^+)	94
7	α-Muurolene	Leaves	30.055	1500	1516	105, 161, 119, 91, 204 (M^+)	95
8	γ-Cadinene	Leaves	30.255	1513	1521	161, 105, 119, 91, 204 (M^+)	96
9	δ-Cadinene	Leaves	30.335	1522	1523	161, 105, 119, 91, 204 (M^+)	97
10	Zonarene	Leaves	30.630	1528	1531	161, 105, 119, 91, 204 (M^+)	93
11	*cis*-Calamenene	Leaves	31.060	1528	1542	159, 202 (M^+), 144, 115, 105	95
12	*trans*-Cadina-1,4-diene	Leaves	31.515	1533	1554	161, 105, 119, 91, 204 (M^+)	92
13	α-Cadinene	Leaves	31.595	1537	1556	161, 105, 119, 91, 204 (M^+)	94
14	Selina-3,7(11)-diene	Leaves	32.005	1545	1566	161, 105, 189, 91, 204 (M^+)	96
15	*trans*-Dauca-4(11),7-diene	Leaves	32.150	1556	1570	161, 105, 119, 91, 204 (M^+)	91
16	Germacrene B	Leaves	32.330	1559	1575	121, 161, 107, 93, 204 (M^+)	93
17	Maaliol	Leaves	32.660	1566	1583	109, 161, 204, 121, 222 (M^+)	92
18	Germacrene D-4-ol	Leaves	32.860	1574	1589	161, 105, 121, 91, 222 (M^+)	94
19	Spathulenol	Bark	35.600	1578	1578	205, 119, 105, 91, 43	98
20	Carotol	Leaves	33.005	1594	1592	109, 122, 161, 93, 222 (M^+)	91
21	Guaiol	Leaves	33.220	1600	1598	105, 161, 204, 119, 222 (M^+)	95
22	Viridiflorol	Bark	35.900	1590	1593	161, 109, 105, 91, 222 (M^+)	96
23	5-*epi*-7-*epi*-α-Eudesmol	Leaves	34.165	1607	1624	161, 105, 204, 91, 222 (M^+)	90
24	10-*epi*-γ-Eudesmol	Leaves	34.980	1622	1646	161, 105, 204, 91, 222 (M^+)	92
25	β-Muurola-4,10(14)-dien-1-ol	Wood/Bark	37.550	1630	1662	161, 105, 204, 119, 222 (M^+)	97
26	Helifolenol A	Leaves	36.015	1674	1674	161, 105, 119, 91, 222 (M^+)	91
27	Eudesm-7(11)-en-4-ol	Leaves	37.310	1700	1709	161, 105, 204, 91, 222 (M^+)	93
28	Amorpha-4,9-dien-2-ol	Leaves	37.565	1700	1717	161, 105, 119, 204, 222 (M^+)	98

R_t: Retention time on HP-5MS column; RI_{calc}: Experimental retention index relative to C_7--C_{30} n-alkanes; RI_{lit}: Reference retention index (Adams, 2017); Key Diagnostic Ions: Main electron impact (70 eV) fragmentation m/z values arranged by relative intensity, with the base peak (100% abundance) in bold; Match Score: Mass spectral similarity percentage obtained against Adams (2017) essential oil spectral library.

Table S2. Chemical traceability, PubChem database identifiers, SMILES strings, InChIKeys, and protonation states of all major essential oil constituents of *Endlicheria paniculata* and reference controls evaluated in molecular docking simulations.

Compound	PubChem CID	Protonation State (pH 7.4)	Canonical SMILES	Isomeric SMILES	InChIKey
**β-Muurola-4,10(14)-dien-1-ol**	102227181	Neutral	CC(C)C1CCC2(C1C=C(C)CC2)O	CC(C)[C@@H]1CC[C@]2([C@H]1C=C(C)CC2)O	MWYFAJHZVOQGCS-SFQUAWSSSA-N
**δ-Elemene**	12306057	Neutral	CC(=C)C1CCC(C(C1)C(=C)C)C=C	CC(=C)[C@H]1CCC@@HC=C	HCYMDYAUCOWBAA-WPVKPHQASA-N
**Tetradecanal**	12534	Neutral	CCCCCCCCCCCCC=O	CCCCCCCCCCCCC=O	NRVSPNBDJJOYRE-UHFFFAOYSA-N
**Isobornyl acetate**	10224	Neutral	CC(=O)OC1CC2CCC1(C2(C)C)C	CC(=O)O[C@H]1C[C@@H]2CC[C@@]1(C2(C)C)C	RKHJRCJMGWSSRO-UJFPJUFBSA-N
**Spathulenol**	92231	Neutral	CC12CCC3C(C1C2(C)C)C(=C)CCC3(C)O	C[C@@H]1[C@H]2CC[C@@]3(C(=C)CC[C@H]13)C2(C)C	WNWLIWDZHKLVDM-XGAUFEBOSA-N
**Viridiflorol**	119245	Neutral	CC12CCC(C1C3C(C2(C)C)CCC3(C)O)C	C[C@@H]1CC[C@H]2[C@@H]1[C@H]3C@@HC2(C)C	SLNVIWIUGRUFI-JIDWCLDDSA-N
**Amorpha-4,9-dien-2-ol**	14385150	Neutral	CC(C)C1CCC2C(=CCC(C2C1)O)C	CC(C)[C@@H]1CC[C@@H]2C	

Table S3. Target proteins, PDB access codes, grid box coordinates (x, y, z), dimensions (AA), and binding free energies (ΔG, kcal/mol) calculated for major constituents of *Endlicheria paniculata* and reference ligands.

Target Enzyme	PDB ID	Grid Center (x,y,z)	Grid Size (A˚)	Compound / Control	ΔG (kcal/mol)
Acetylcholinesterase (AChE)	4EY1	$x = 14.2, y = -22.5, z = 11.8$	$20 \times 20 \times 20$	Viridiflorol	-7.4
				Selina-3,7(11)-diene	-7.2
				Spathulenol	-6.9
				Neostigmine *(Control)*	-8.2
α-Glucosidase	3A4A	$x = 21.5, y = -0.8, z = 9.3$	$22 \times 22 \times 22$	Amorpha-4,9-dien-2-ol	-6.8
				β-Muurola-4,10(14)-dien-1-ol	-6.5
				Acarbose *(Control)*	-8.1
